# Immunoglobulin G structure and rheumatoid factor epitopes

**DOI:** 10.1371/journal.pone.0217624

**Published:** 2019-06-14

**Authors:** Sheila Lefoli Maibom-Thomsen, Nicole Hartwig Trier, Bettina Eide Holm, Kirsten Beth Hansen, Morten Ib Rasmussen, Anna Chailyan, Paolo Marcatili, Peter Højrup, Gunnar Houen

**Affiliations:** 1 Department of Biochemistry and Molecular Biology, University of Southern Denmark, Odense, Denmark; 2 Department of Autoimmunology, Statens Serum Institut, Copenhagen, Denmark; 3 Department of Bioinformatics, Technical University of Denmark, Kongens Lyngby, Denmark; New York State Department of Health, UNITED STATES

## Abstract

Antibodies are important for immunity and exist in several classes (IgM, IgD, IgA, IgG, IgE). They are composed of symmetric dimeric molecules with two antigen binding regions (Fab) and a constant part (Fc), usually depicted as Y-shaped molecules. Rheumatoid factors found in patients with rheumatoid arthritis are autoantibodies binding to IgG and paradoxically appear to circulate in blood alongside with their antigen (IgG) without reacting with it. Here, it is shown that rheumatoid factors do not react with native IgG in solution, and that their epitopes only become accessible upon certain physico-chemical treatments (e.g. heat treatment at 57 °C), by physical adsorption on a hydrophobic surface or by antigen binding. Moreover, chemical cross-linking in combination with mass spectrometry showed that the native state of IgG is a compact (closed) form and that the Fab parts of IgG shield the Fc region and thereby control access of rheumatoid factors and presumably also some effector functions. It can be inferred that antibody binding to pathogen surfaces induces a conformational change, which exposes the Fc part with its effector sites and rheumatoid factor epitopes. This has strong implications for understanding antibody structure and physiology and necessitates a conceptual reformulation of IgG models.

## Introduction

### Immunoglobulins

**Fig 1:**
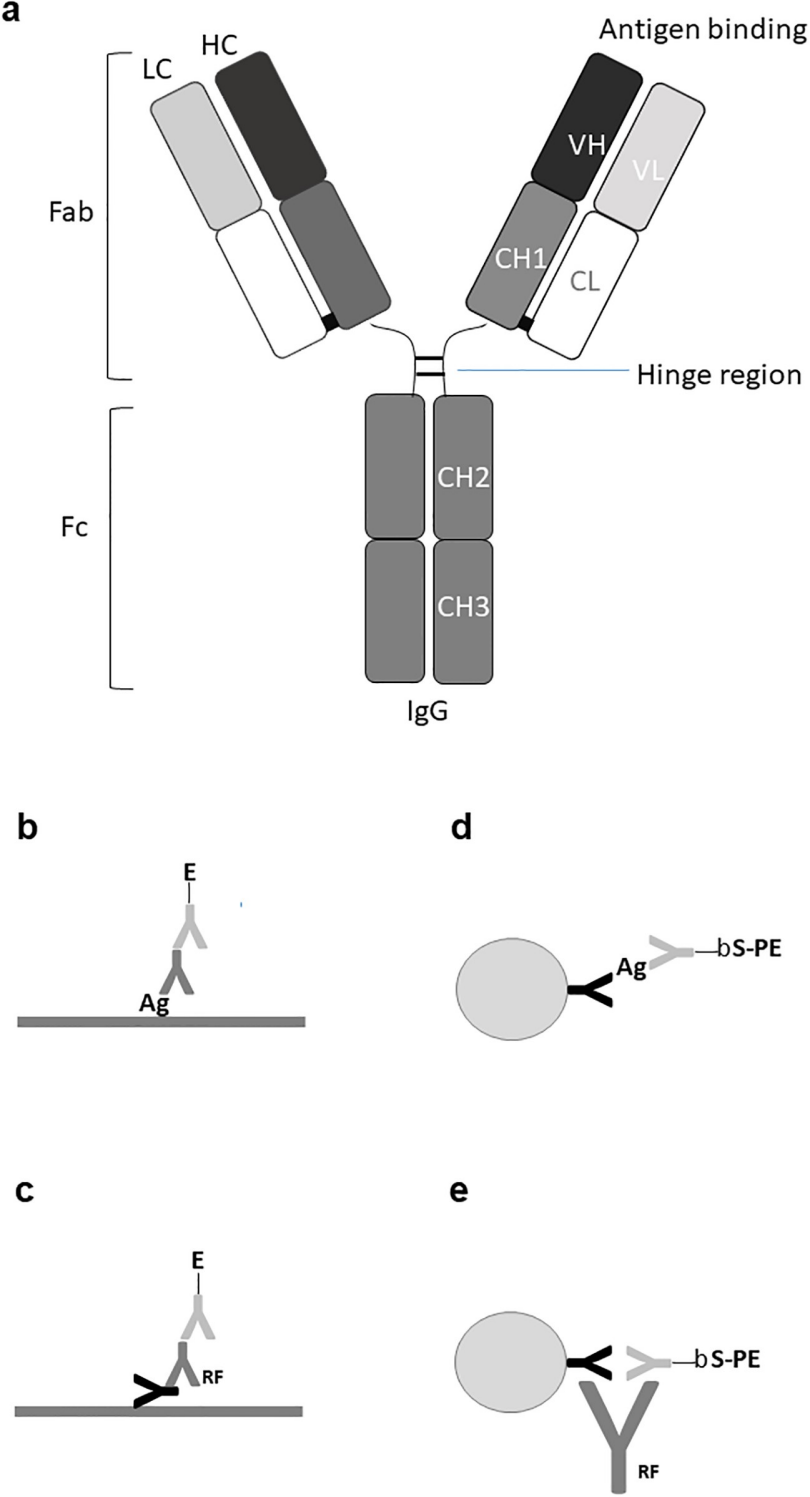
Immunoglobulin model and immunoassay formats used in this work. (**a)**. Schematic structure of an immunoglobulin G (IgG) molecule consisting of two heavy chains of γ type and two light chains of either κ or λ type linked by disulphide bridges. The variable part of an heavy chain together with the variable part of a light chain together form the antigen binding site. A light chain together with variable heavy and CH1 domains form a Fab (fragment antigen-binding) part (“arm”) and the CH2 and CH3 domains constitute the Fc (fragment constant or crystallizable) part with effector functions. (**b)**. Direct antigen antibody ELISA, where the antigens are immobilized by non-covalent forces. (**c)**. Rheumatoid factor (RF) ELISA, where the antigen is IgG. (**d)**. Bead-based fluorescent capture sandwich immunoassay, where the capture antibody is immobilized by covalent bonds. (**e**). RF sandwich/bridging assay. Ab: antibody, Ag: antigen, E: enzyme, b: biotin, S: streptavidin, PE: phycoerythrin, RF: rheumatoid factor. CL: constant light, VL: variable light, VH: variable heavy, CH: constant heavy.

Immunoglobulins (Igs) constitute an important part of the immune defence against pathogens and help to recognize and remove foreign antigens [1–4]. Igs are also called antibodies and occur in several classes (IgM, IgD, IgA, IgG, IgE) and subclasses (IgG1-4, IgA1,2) with different structures and effector functions. However, all Igs/antibodies share the same basic unit design of two identical heavy chains with an N-terminal variable domain, and three or four constant domains (CH1-CH4) and two identical light chains with an N-terminal variable domain and a C-terminal constant domain, all linked by disulphide bonds and usually depicted schematically as in Fig 1a [1–4]. The heavy chains can be of different types (classes) called μ, δ, α, γ, ε, corresponding to IgM, IgD, IgA, IgG, IgE, while the light chains can be of either κ or λ type. The variable N-terminal domains of the light and heavy chains together form two antigen-binding sites and together with the CH1 and constant light domains, these form the parts (arms) known as fragment antigen-binding (Fab). The CH2 and CH3 (CH2-4 in IgM and IgE) domains together form a part designated fragment crystallisable (or constant) (Fc) (Fig 1a) [1–4].

### Rheumatoid factors and immunoassays

Rheumatoid factors (RFs) are autoantibodies recognizing the Fc part of other Igs. They are mainly found in blood samples from patients with rheumatoid diseases (e.g. rheumatoid arthritis (RA)) and occur in different forms, but the most prominent are IgM and IgA RFs binding to the Fc part of IgG [5–8]. RFs can be measured by various immunoassays, including enzyme-linked immunoassay (ELISA), where the antigen is coated in wells of a microtitre plate. Fig 1b illustrates a direct ELISA, where antigen coated on the surface of a microtitre well has reacted with a primary antibody from a patient serum sample, which has subsequently reacted with an enzyme-labelled secondary antibody (conjugate). This type of assays can also be performed with antigen covalently immobilized on fluorescent beads (fluorescence-linked immunosorbent assay, FLISA). In the case of RFs, the antigen is itself an antibody (IgG) (Fig 1c), necessitating the use of IgM- and IgA-specific secondary enzyme-conjugated antibodies (conjugates) for detection/quantification. For some purposes, it is advantageous to use an immoblized antibody to capture an antigen and then detect the bound antigen with a second antibody, having specificity for a non-overlapping epitope on the same antigen. Such assays are called capture or sandwich assays and can be performed in ELISA format or as bead-based assays with a covalently immobilized capture antibody and a fluorescence-labelled secondary detecting antibody, or as shown in Fig 1d, a biotin-labelled detecting antibody, which can be quantified with fluorescence-labelled streptavidin. Such “sandwich” assays are sensitive to the presence of RFs, since RFs may “bridge” the capture and the detecting antibody in the absence of antigen giving rise to false positive results (Fig 1e) [5].

### Rheumatoid factor epitopes and immunoglobulin structure

The epitopes of RFs have been characterised in several cases and reside in the Fc part of IgG, often in the CH2/CH3 groove or the CH3/CH3 groove (Fig 1a) [9–12]. Since RFs can be measured in sera from RA patients and as the concentration of IgG in serum is roughly 5–10 times higher than the concentration of IgM and IgA [1], an apparent paradox is how the RFs can circulate in blood of patients with rheumatoid diseases alongside their antigen (IgG) in large amounts without reacting with it and being cleared from the circulation. Here, we show that native IgG has a compact, closed structure, where the binding sites for the majority of RFs are shielded by the Fab arms and only become exposed upon antigen binding or by certain physicochemical treatments, which induce a conformational change in IgG similar to that seen upon antigen binding. RF epitopes can therefore be regarded as cryptic epitopes and this resolves the apparent paradox of how RFs can circulate in high concentrations in parallel with IgG in patients with rheumatic diseases, since the binding sites on IgG for RFs are only exposed upon antigen binding.

## Results

### Rheumatoid factors circulate in parallel with their antigen, IgG, in serum

In order to fully illustrate the paradoxical situation with RFs being present in sera from RA patients alongside an excess of their antigen, IgG, we performed a gelfiltration experiment with a pooled sample of RF-positive sera (S1 Fig). This revealed that IgM eluted before IgA, which eluted before IgG in accordance with their different molecular masses, IgM being a pentamer of dimers, IgA a dimer (of dimers) and IgG a dimer [1]. Also, RF IgM and RF IgA were measurable in the fractions from the RF-positive sample (whereas no RF activity was present in a healthy donor (HD) pool). This clearly illustrates the paradoxical situation with RFs being present in RA sera without reacting with their antigen, IgG, since no IgG was present in the IgM peak and since the RFs in the peak were capable of reacting with IgG in the RF ELISA.

Furthermore, when an RA sample pool was incubated with heat-treated IgG, a white precipitate was formed, which was not seen in a parallel healthy donor (HD) sample pool (S2 Fig). The precipitate was found by SDS-PAGE and immunoblotting to contain IgM, IgA and IgG, most likely from RF IgM/IgA complexes with IgG (S2 Fig). The remaining, non-precipitated RA sample pool supernatant and the HD sample pool (with added heat-treated IgG) were analysed by gelfiltration chromatography (S3 Fig), which showed that IgM, IgA and IgG eluted sequentially in both samples, but that elution profiles of the Igs were broader for the RA sample pool. When fractions were analysed for RFs, none were seen in the HD sample, and a faint RF IgM activity but no RF IgA was seen for the RA sample pool. This indicated that most RFs had precipitated in the RA sample pool upon incubation with heat-treated IgG, but that some complexes of RFs and IgG remained soluble, giving rise to broader Ig peaks and to a faint RF IgM activity from RF IgM with remaining free antigen-binding sites.

The results described above indicated that RFs circulate in parallel with their antigen, IgG, in serum without reacting with it. This was also illustrated by an ELISA experiment, where total IgM from RA and HD sample pools were captured by antibodies to IgM and then exposed to IgG, which had first incubated overnight at 5 °C or 57 °C (S4 Fig). The captured RA IgM, containing RFs IgM, was found to interact with heat-treated IgG but not with control IgG, whereas no interaction was seen for the HD sample pool IgM.

### Rheumatoid factors do not react with IgG in solution

**Fig 2:**
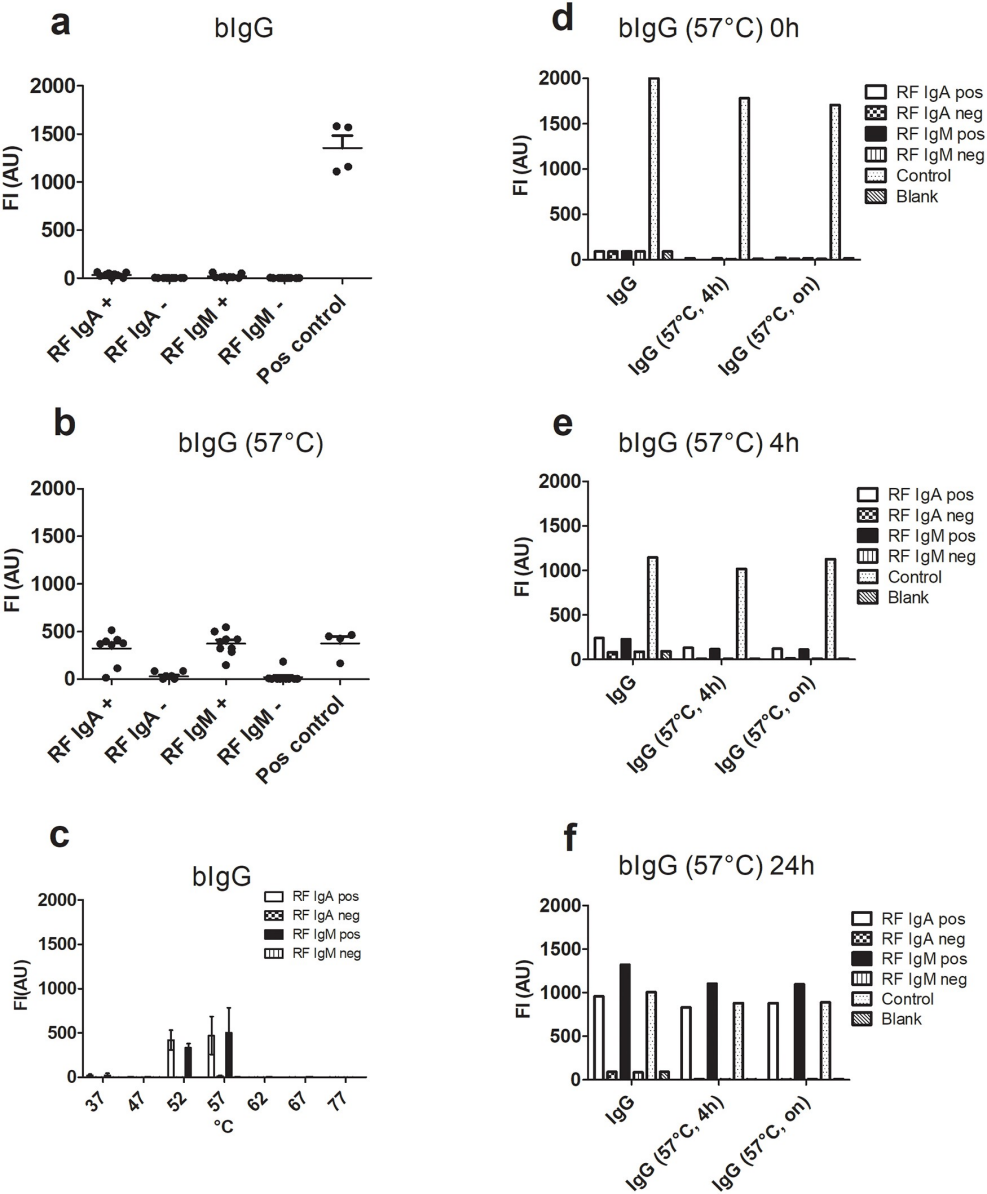
RF reactivity with IgG. RFs do not react in a bead-based fluorescent sandwich/bridging immunoassay with immobilized IgG and bIgG in solution but do so after exposure of the bIgG to elevated temperature. (**a)**. Reactivity of RF-positive and -negative sera to IgG immobilized on fluorescent beads (positive control: RaHIgG). Individual data points represent single determinations on nine individual sera. **(b)**. RFs react with heat-treated (57 °C, 24 h, heating cabinet) bIgG (***: p = 0.0003 for RF IgA, p = 0.0001 for RF IgM). Individual data points represent single determinations on the same nine sera as used in (**a**). The figures in (**a**,**b)** show one representative experiment of three. **c**. Temperature dependence of RF reactivity. IgG incubated at the indicated temperatures was tested for reaction with RF-positive or–negative sera using beads with immobilized IgG. The figure shows mean +/- SD of four experiments. (**d-f)**. Native IgG (0 h) and IgG incubated at 57 °C for 4 h or 24 h was covalently immobilized on fluorescent beads and incubated with RF-positive or -negative serum samples in the presence of bIgG, which was either non-heated (**d**, 0 h) or had been incubated at 57 °C for 4 h (**e**) or 24 h (**f**). The panel shows one of two experiments. Individual data points of the time experiment are single determinations on individual sera (4 in all). The positive control was rabbit antibodies to human IgG (RaHIgG).

As mentioned above, assays for RFs are routinely done by ELISA with immobilized (protein A affinity-purified) IgG (Fig 1c). RFs can also be measured by nephelometry or agglutination assays, which are performed with heat-aggregated IgG (30–60 min, 63 °C) [5,13–17]. In this work, we attempted to develop a multiplex RF assay based on fluorescent multiplex bead technology, taking advantage of the ability of RFs to bridge immobilized IgG with (labelled) IgG in solution (Fig 1e). To our great surprise, these experiments showed that sera having either IgM RF, IgA RF or both (as measured by the ELISA method (Fig 1c)) did not react in a sandwich/bridging assay with ion exchange (IEX)-purified human IgG (covalently immobilized on beads through amide bonds with lysine side chains) and biotinylated IgG (bIgG) as the detecting agent (Fig 2a).

In the bead-based sandwich assay, human IgG was immobilized on fluorescent beads, and the beads were then incubated with human sera and bIgG together (bridging assay) followed by detection of bead-bound bIgG with phycoerythrin (PE)-labelled streptavidin (S). In principle, any RF present would be capable of bridging bead-bound IgG and bIgG in solution, provided that the immobilized IgG and the bIgG adopted RF-binding conformations (Fig 1e). A a positive control, rabbit Igs against human IgG (RaHIgG) gave a strong reaction, confirming that IgG had indeed been immobilized and could be bridged to bIgG in solution, thus verifying the viability of the assay (Fig 2a). Since these results were obtained with sera, which had been shown to contain RFs by routine ELISA with immobilised protein A-purified IgG, we confirmed that the sera also showed RF reactivity with the IEX-purified IgG in the routine ELISA, in which antigens (in this case IgG) are passively adsorbed (coated) on a hydrophobic polystyrene surface (S5a Fig). As part of the multiplexing assay, we also tested for a possible reaction of RFs with IgM and IgA. As expected, no reaction of RFs was observed when using covalently immobilised human IgM or IgA and bIgG as detecting agent (S6a–S6c Fig). In conclusion, RFs do not react with IgG in solution but react with covalently immobilised IgG and IgG passively adsorbed on a hydrophobic surface.

### A conformational change in IgGs allows for RF interaction

From the experiments described above, we hypothesized that the RF epitopes could be hidden in the IgG structure (cryptic epitopes) and only become exposed by conformational changes, which may occur upon antigen binding *in vivo* or *in vitro*, upon immobilization on surfaces (e.g. the hydrophobic polystyrene surface used in routine ELISA for RFs) or by certain physico-chemical conditions (e.g. elevated temperature).

This prediction was verified by subjecting the immobilized IgG and/or the soluble bIgG to elevated temperatures (Fig 2b–2f). This revealed that the covalently immobilized IgG was capable of RF interaction and could be bridged to bIgG heat-treated at 57 °C (Fig 2b). Moreover, the soluble bIgG did not interact with RF, but acquired the ability to be bound by RF in a time-dependent manner upon incubation at 57 °C (Fig 2d–2f). Incubation at 52 °C and 57 °C gave rise to interaction, whereas 47 °C did not induce the conformational change necessary for RF binding and 62 °C resulted in diminished/abolished interaction (Fig 2c). This temperature profile was observed both in the bridging FLISA (Fig 2c) and in a similar bridging ELISA (S5b Fig). Moreover, the heat-treated IgG was capable of RF interaction, when coated in polystyrene microtitre wells up to about 60 °C. The absence of RF interaction above 60 °C was due to more extensive structural changes and/or precipitation of the IgG but not to denaturation, since unfolding of the IgG (measured by an Eva Green fluorescence assay [18]) showed a broad peak corresponding to T_m_ values of 70–80 °C (S7 Fig). Whereas heat-treated IgG acquired the ability to bind RFs, no interaction of RFs with heat-treated IgM and IgA was seen thus verifying the specificity of the assay (S6d–S6f Fig).

As described above, the IEX-purified IgG adsorbed on the polystyrene surface of ELISA plates at room temperature acquired the ability to bind RFs similar to protein A-purified IgG, showing that the physical interaction with the hydrophobic surface induces a conformational change allowing RF binding. In agreement with this, not only did the heat-treated IgG adsorbed directly on the polystyrene surface exhibit similar RF binding as the adsorbed native (non-heated) IgG (S5a Fig), the heat-treated IgG also retained the ability to bind protein G and protein A (S5c–S5e Fig). Moreover, in the bridging ELISA assay with native IgG immobilized at room temperature, the heat-treated bIgG (captured by the bridging RFs) showed binding if pre-incubated at 47–57 °C (S5b Fig) as also observed in the bead-based assay with immobilized IgG (Fig 2b–2f). Finally, when the proteins in the RF-containing sera (including the RFs) were themselves coated directly on the polystyrene surface of ELISA plates, they bound soluble bIgG optimally, if it had been incubated at 57 °C first (S5f Fig), as also seen by capture ELISA (S4 Fig).

**Fig 3:**
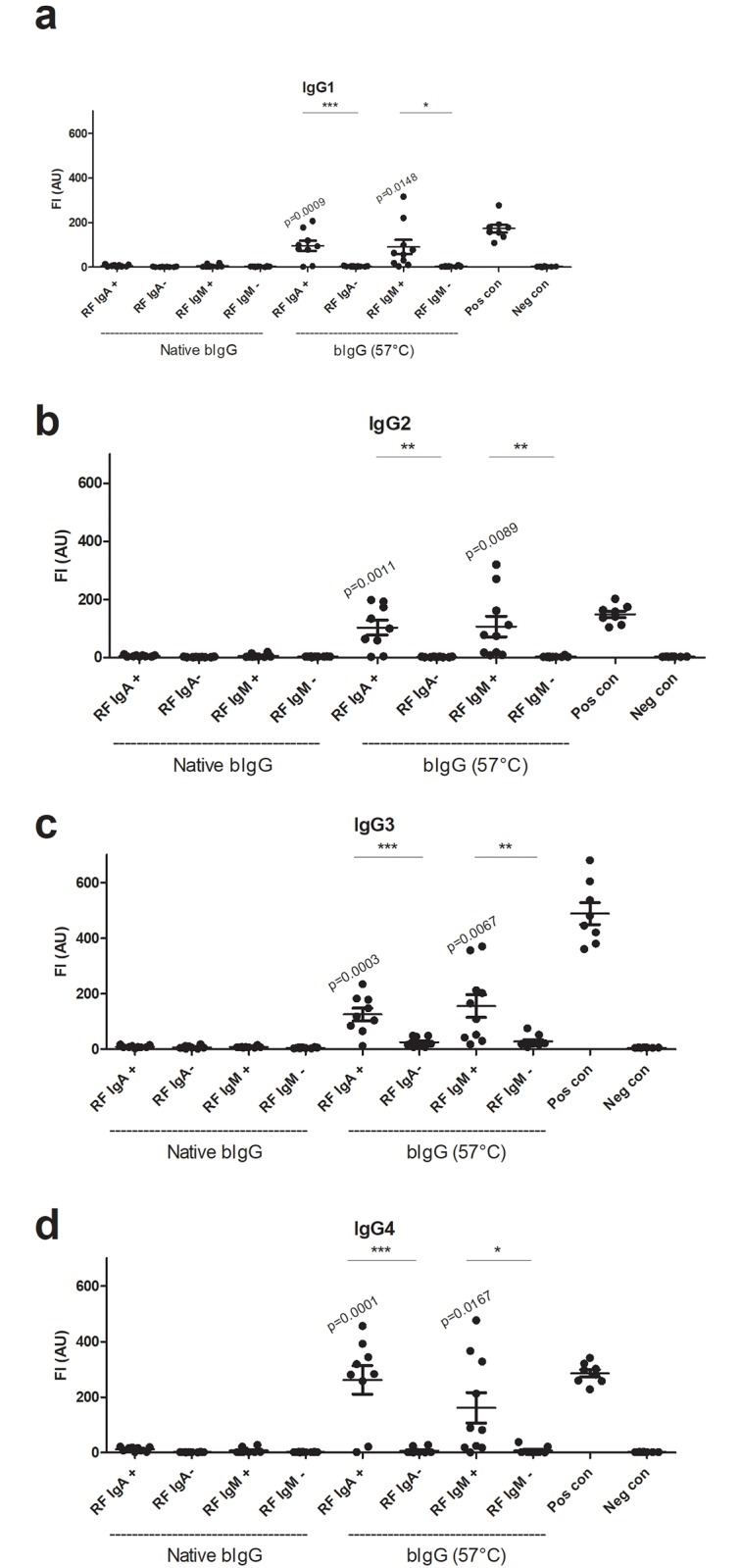
RF reactivity with IgG subclasses. RFs do not react with native IgG in a bead-based fluorescent sandwich/bridging immunoassay with immobilized IgG subclasses but does so after exposure of the bIgG to elevated temperature (57 °C, 24 h). (**a**). IgG1. (**b**). IgG2. (**c**). IgG3. (**d**). IgG4. The figures show one of two experiments.

The experiments described above were conducted using a natural mixture of human IgGs (i.e. intravenous immunoglobulin (IVIG)), highly purified by IEX chromatography and primarily containing IgG1 (60%, 33%, 3% and 2% of IgG1, IgG2, IgG3 and IgG4, respectively) [19]. To investigate whether this picture only relates to IgG1 or to all IgGs, purified IgG1, IgG2, IgG3 and IgG4 were coupled to microsperic beads and analysed for RF reactivity. Independent of the IgG subtype, reactivity with RFs was found when the bIgG had been pre-incubated at 57 °C (Fig 3).

Due to the surprising results obtained in the assays described above, we carried out further experiments to verify the conclusions implicated by the results, using both the natural IgG preparation and monoclonal IgG products.

**Fig 4:**
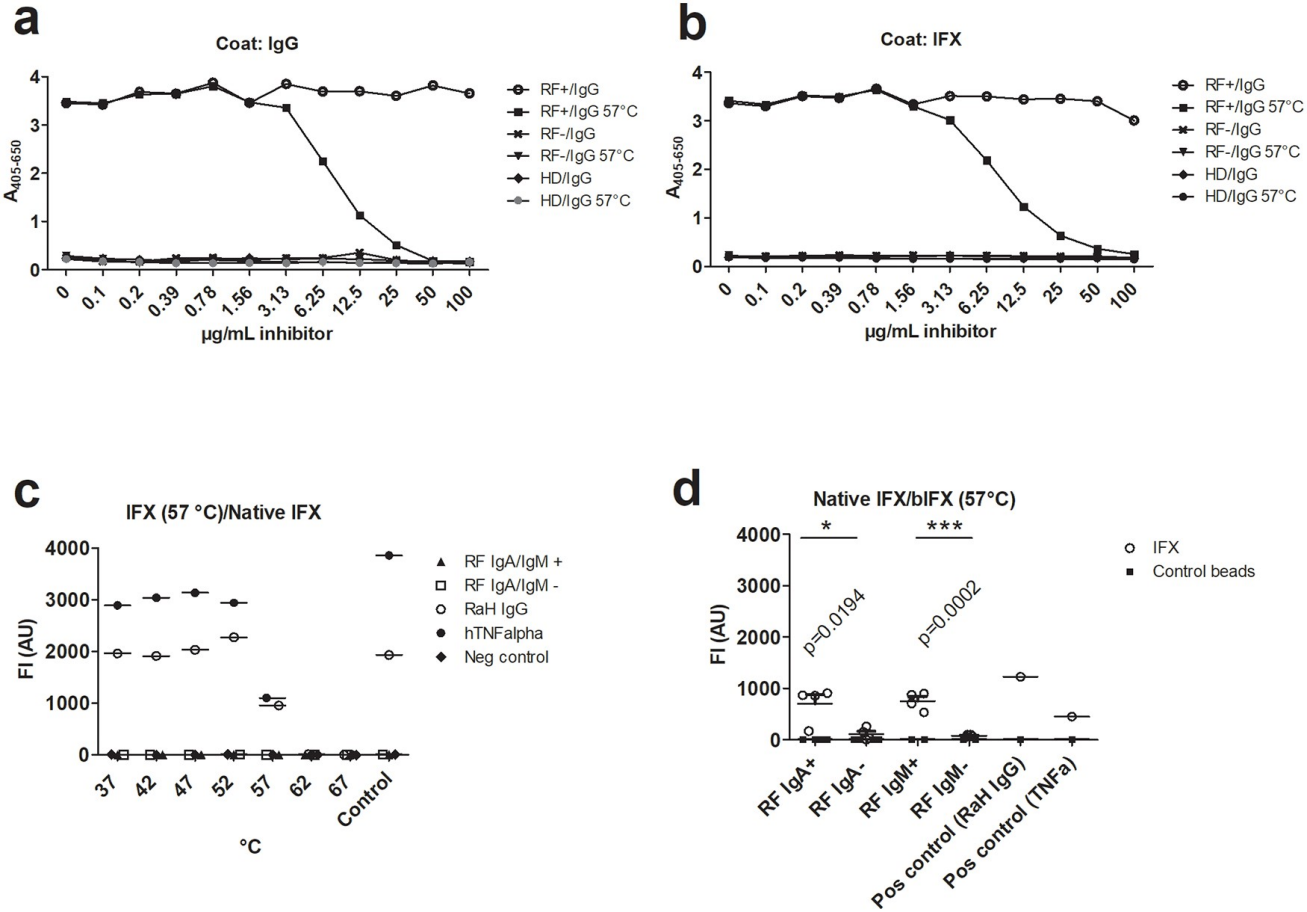
IgG reactivity with RFs. (**a, b)**. Reaction of different IgG forms with Rheumatoid factors in inhibition assays. IgG (**a**) or IFX (**b**) was coated on the surface of polystyrene ELISA plates and incubated with RF-containing serum or control serum (healthy donor serum) in the absence or presence of the indicated concentrations of inhibitor (native or heat-treated IgG). The experiments were done as titrations and data points are single determinations. (**c**). Immobilised IFX incubated first at 37 °C—57 °C and then immobilized on beads reacts with RaHIgG and can be bridged to native IFX in solution by TNF but not by RFs. No reaction is seen with IFX incubated at 62 °C or 67 °C due to precipitation of the IFX. (**d**). IFX immobilized covalently on beads can be bridged by RFs to heat-treated IFX (57 °C) but not by RF-negative sera. The figures show one representative experiment out of two. HD: healthy donors (pool).

Inhibition experiments (Fig 4) verified the conclusions reached by the solid-phase assays, as heat-treated IgG was capable of inhibiting RF binding to immobilized IgG, whereas native IgG showed no inhibition (Fig 4a). Moreover, the monoclonal therapeutic IgG Infliximab (IFX), directed to tumor necrosis factor (TNF), showed identical behavior when substituted for IgG on the solid phase (Fig 4b), and elution experiments confirmed the reaction of RFs with both “natural” IgG (IVIG) and IFX, since RFs eluted from IVIG reacted with IFX and vice versa (S8 Fig).

Overall, experiments with IFX showed identical behavior as observed with IgG (IVIG), except that the monoclonal IFX changed conformation at a somewhat lower temperature and had a tendency to precipitate at 57 °C (Fig 4c and 4d, S9a Fig). As seen in Fig 4c, native biotinylated IFX (bIFX) could not be bridged to immobilized IFX, irrespective of heat treatment of the latter. Notably, both the trimeric TNF and the RaHIgG could bridge the immobilized IFX and the soluble bIFX, albeit with a reduction/abolishment in signal, when the IFX had been treated at 57 °C or 62–67 °C, respectively (due to precipitation of IFX). In contrast, with the reverse configuration, i.e. immobilization of native IFX and incubation with sera in the presence of heat-treated IFX (57 °C), bridging by RFs was observed (Fig 4d). This verified the results obtained with the IEX-purified IgG, showing that immobilized IFX and heat-treated IFX both obtained an “open”, RF-interacting conformation. The same conclusion was reached by ELISA, showing that immobilized (coated) IFX could be bridged to IFX in solution, provided the latter had been incubated first at 51–57 °C (S9a Fig) and that this reaction could be inhibited by heat-treated (57 °C) IgG (S9b Fig). Nivolumab and Ipilimumab, two other therapeutic monoclonal antibodies (directed to programmed death (PD) and cytotoxic T lymphocyte-associated protein 4 (CTLA4)), and Enbrel (a tumor necrosis factor receptor (TNFR)-Fc construct), when coated in ELISA wells, showed reaction with RFs in a manner that could be inhibited by heat-treated IgG (S9b Fig). This showed that human Fc-containing molecules displayed RF epitopes upon adsorption to the hydrophobic polystyrene surface. Moreover, several murine monoclonal antibodies exhibited identical behavior, showing that the murine IgG Fc also reacts with RFs upon exposure (S9b Fig) in agreement with their ability to cross-react with RFs [5].

### Heat-treated IgG retains antigen-binding ability and antigen binding exposes cryptic RF epitopes

**Fig 5:**
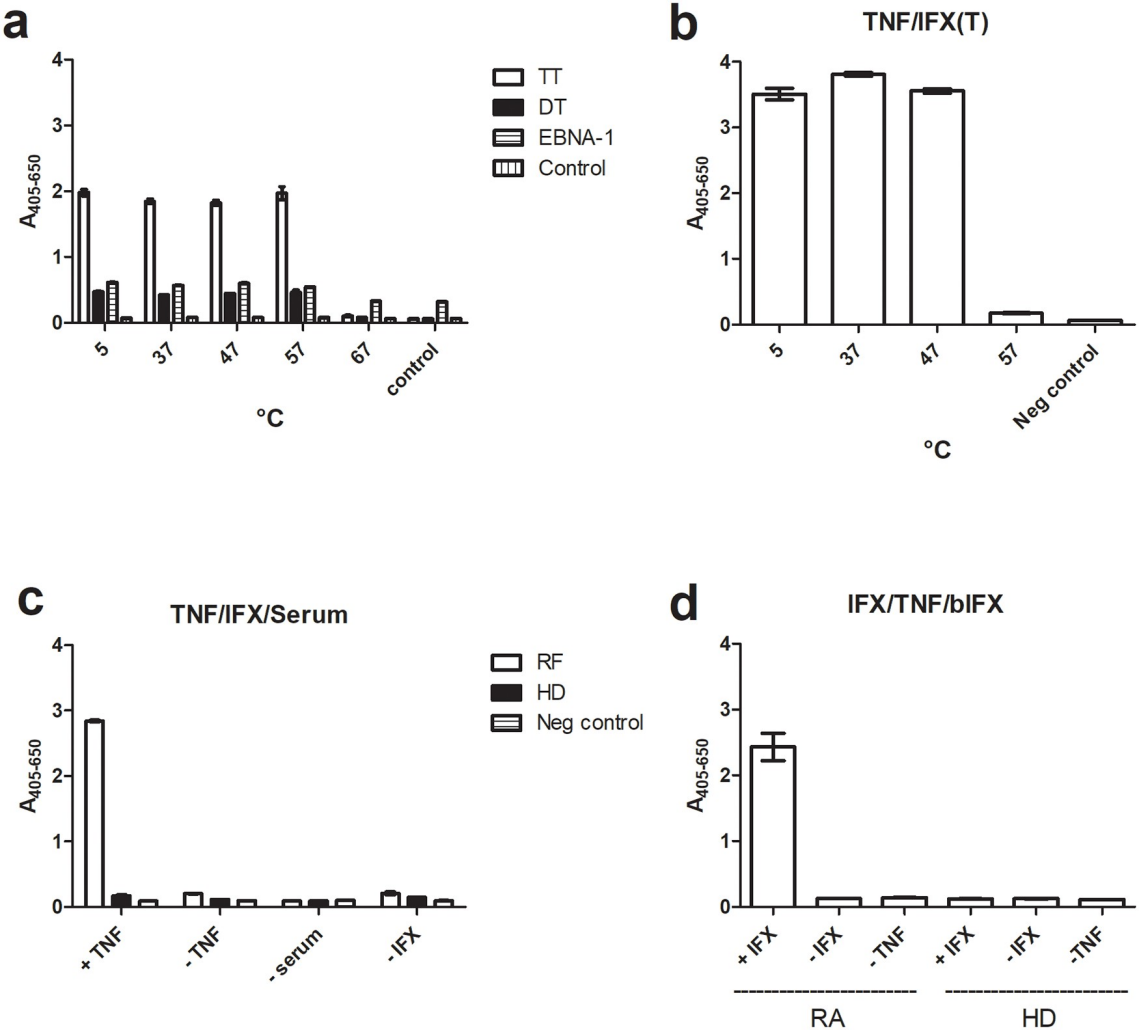
Heat-treated IgG retains antigen binding and antigen binding exposes cryptic RF epitopes. (**a**). Tetanus toxoid (TT), diphtheria toxoid (DT) and Epstein Barr virus nuclear antigen 1 (EBNA1) were coated in ELISA wells and incubated with bIgG (bIVIG), which had been pre-incubated at the indicated temperatures. (**b**). TNF was coated in ELISA wells and incubated with bIFX, which had been pre-incubated at the indicated temperatures. (**c**). TNF was immobilized on the surface of a microtitre plate, incubated with IFX and then incubated with RF-positive or -negative serum. (**d**). Infliximab was immobilized in ELISA wells and incubated with an RF-positive serum, which had first been incubated with IFX (which had itself been pre-incubated with TNF). The figures show one experiment out of two. HD: healthy donors (pool).

Denaturation of IgG would be expected to impair antigen binding, whereas less drastic conformational changes involving the relative orientation/location of the IgG Fab arms and the Fc could very well retain antigen binding ability. Heat-treated IgG (up to 57 °C) was capable of binding various antigens (tetanus toxoid (TT), diphtheria toxoid (DT) and Epstein Barr Virus nuclear antigen 1 (EBNA 1) (Fig 5a), and heat-treated IFX retained the ability to bind TNF but was somewhat less tolerant to incubation at 57 °C due to precipitation (Fig 5b). This strongly indicated that the conformational changes upon heat treatment up to about 57 °C only affected the relative orientation of the Fab and Fc parts.

As described, IFX immobilized on beads and incubated with (biotin-labelled) IFX (bIFX) in the presence of RFs showed no binding unless the bIFX had been heat-treated (Fig 4c and 4d). Similarly, IFX immobilized on the polystyrene surface of ELISA plates could only be bridged by RFs to bIFX in solution, if this had been heat-treated (S9a Fig).

When IFX was allowed to bind solid phase-adsorbed TNF, the IFX adopted an RF-interacting conformation (Fig 5c). Moreover, when IFX was adsorbed to the surface, it could be bridged by RFs to bIFX in solution, if the soluble bIFX had been pre-incubated with TNF, which itself can bridge IFX molecules and also Fab “arms” on the same molecule due to its trivalency. This indicated that TNF bridging of “neighbour” Fab arms on individual IFX molecules induced an RF-binding conformation (Fig 5d).

### A conformational change in IgG increases interaction with ConA and C1q

Due to the surprising and unexpected results with RF interaction with IgG, we decided to investigate the interaction of IgG with other Fc-interacting molecules and to test the dependency on heat treatment.

The carbohydrate structures on IgG are N-linked glycans [1], which might also be at least partially shielded by the Fab arms due to their location in IgG. Actually, when IgG was immobilized on protein G, the heat-treated IgG showed increased binding of ConA relative to the native IgG (S10a Fig) as an indication of a conformational change (“opening”) facilitating access to the structural carbohydrate.

The ability of IgG to activate the classical complement pathway is well known [1]. However, it has remained partly unanswered, how C1(q) can exist in parallel with IgG in circulation, a paradoxical situation similar to the co-circulation of IgG and RFs. When C1q was coated on the surface of ELISA plates, it did not show interaction with bIgG in solution, unless the bIgG had been subjected first to elevated temperature, an effect that was maximal at 57 °C (S10b Fig).

### Accessibility of the IgG hinge region and RF specificity

Due to the structural implications of the results, the question arose, whether the hinge regions in “closed” and “open” IgG would be equally accessible to proteolytic cleavage. Therefore, Fab and Fc fragments of human IgG were generated by cleavage with the hinge-specific IdeS protease [20]. No significant differences in cleavage pattern were seen for IgG and heat-treated IgG, showing that the hinge region is accessible in both native “closed” and in “open” IgG (S11a and S11b Fig).

Furthermore, using the intact and cleaved IgG in Western immunoblotting, we confirmed the specific reaction of RFs with the Fc part of IgG (S11c and S11d Fig).

### Chemical cross-linking and molecular modelling of native IFX reveal a closed, compact structure

**Fig 6:**
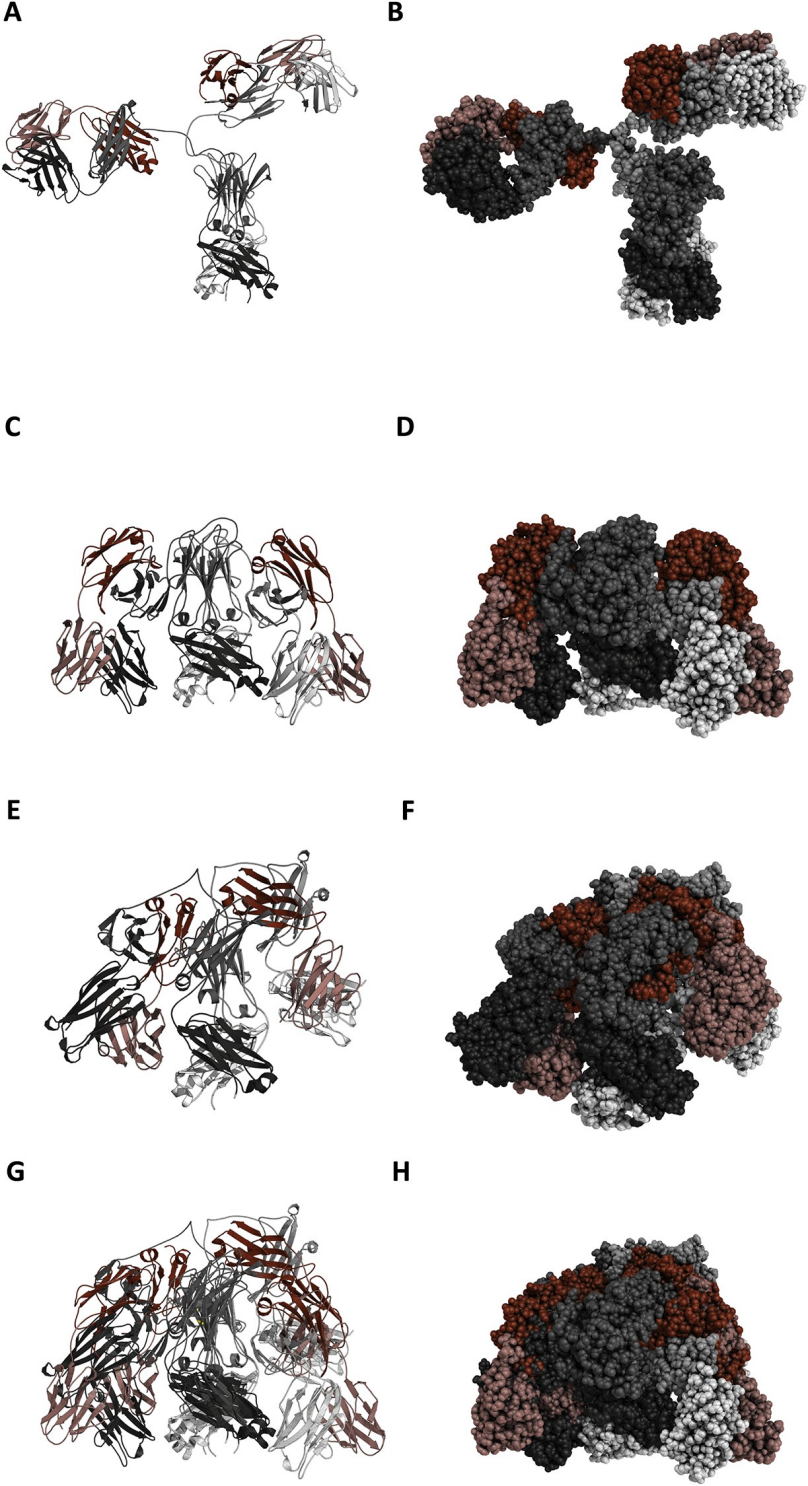
Models of the open and closed IgG (IFX) conformation constructed by Modeller^27^ and using the PDB structures 5VH4, 5VH5 and 1IGT as templates. Left side: ribbon (cartoon) presentation, right side: sphere (space-filling) presentation. Light chains are colored in shades of red, heavy chains in shades of grey. (**a, b)**. Model of the open conformation of the IFX antibody. (**c, d)**. Model of the first closed conformation. (**e, f)**. Model of the second closed conformation. (**g, h)**. Superimposition of first and second closed models.

Modeller [21] was used to build a three-dimensional structure of a traditional “open” conformation of the IFX antibody. An initial model of the classical Y-shaped IFX (open conformation) was constructed using 5VH4, 5VH5 and 1IGT PDB structures as templates for the Fc, Fab, and overall conformation, respectively. The sugars were modelled using the ones present in the PDB structure 5VH5 (Fig 6).

To establish the actual structure of the folded, native IgG, we performed chemical crosslinking. IFX samples were incubated for 2 hours at 37 °C, prior to chemical crosslinking. The homobifunctional chemical crosslinker bis-sulfosuccinimidylsuberate (BS3) targets primary amines, i.e. the ε-amine group of lysine residues and the α-amino group of the protein N-terminus. BS3 has a spacer-arm length of 11.4 Å, and residues within appropriate distance may be covalently linked to each other (<30Å for Cα-Cα [22]). Two separate crosslinking experiments were performed, only varying in the concentration of IXF during crosslinking (Experiment 1: 0.5μg/μL, Experiment 2: 1μg/μL). After crosslinking, the samples were separated by SDS-PAGE, and the gel bands corresponding to the mass of a monomeric antibody were excised from the gel and digested using trypsin (S12 Fig). The resulting peptides were examined by liquid chromatography tandem mass spectrometry (LC-MS) using a Q-Exactive HF mass spectrometer (S13 Fig). Data analysis was performed using the in-house developed software MassAI version April 2018 [23].

Only MS/MS spectra representing cross-links with a score higher than 12 were accepted and all were manually verified. A total of 20 crosslinks were found and are presented in Table 1. Data analysis was approached by considering the open IgG (Y-shaped) structure and dividing the obtained crosslinks into either a ‘validating’ category or an ‘overlength’ category. The validating category contains crosslinks located within crosslinking distance (Cα-Cα <30Å) based on the open IgG model in Fig 6, i.e generally between residues in the same or neighbouring domains. The overlength category contains crosslinks between residues in different domains and with Cα-Cα distances larger than 30Å. The two crosslink experiments produced overlapping sets of overlength crosslinks. Thus, two separate closed conformation antibody models were generated. Model 1 was based on overlength crosslinks determined in Experiment 1, and Model 2 was based on overlength crosslinks from Experiment 2. One crosslink was determined in both experiments, and both models satisfy the distance requirements for all four overlength crosslinks determined in the respective experiments. As a control, native IFX was digested with trypsin and analyzed by LC-MS using the same settings as for the crosslinked samples. The resulting sequence coverage was 78.1% for the heavy chain and 64.4% for the light chain, and no crosslinks were found in the control digest.

**Table 1:** Crosslinks found from validating and overlength categories.

**Validating crosslinks**											
**Domain A**	**Res A**	**Domain B**	**Res B**	**Peptide A**	**Peptide B**	**Exp. 1**	**Exp. 2**	**Model 1**	**Model 2**		**Distance open**
VH	43	VL	49	QSPEKGLEWVAEIR	LLIKYASESMSGIPSR		✖	21.73	22.7		21.71
VH	43	VH	54	QSPEKGLEWVAEIR	SKSINSATHYAESVK		✖	33.17	30.66		32.57
VH	54	VH	78	SKSINSATHYAESVK	DDSKSAVYLQMTDLR	✖		14.26	13.3		14.01
CH1	136	CH1	217	GPSVFPLAPSSKSTSGGTAALGCLVK	KVEPK	✖	✖	18.86	19.66		19.04
CH2	277	CH2	325	TPEVTCVVVDVSHEDPEVKFNWYVDGVEVHNAK	CKVSNK		✖	8.21	8.43		8.08
CH2	320	CH2	343	VVSVLTVLHQDWLNGKEYK	AKGQPR	✖	✖	9.17	8.85		10.3
CH2	337	CH2	343	ALPAPIEKTISK	AKGQPR	✖		15.66	19.32		16.8
CH3	363	CH3	417	EPQVYTLPPSRDELTKNQVSLTCLVK	LTVDKSR		✖	7.24	7.32		7.44
CH3	395	CH2	343	GFYPSDIAVEWESNGQPENNYKTTPPVLDSDGSFFLYSK	AKGQPR		✖	23.71	19.86		23.17
VL	1	VH	43	DILLTQSPAILSVSPGER	QSPEKGLEWVAEIR	✖	✖	19.72	13.48		20.21
VL	1	VL	49	DILLTQSPAILSVSPGER	LLIKYASESMSGIPSR		✖	19.78	21.16		20.25
VL	1	VH	54	DILLTQSPAILSVSPGER	SKSINSATHYAESVK	✖	✖	27.14	30.2		27.54
VL	1	VH	67	DILLTQSPAILSVSPGER	SINSATHYAESVKGR	✖		16.3	17.39		16.06
**Overlength crosslinks**											
**Domain A**	**Res A**	**Domain B**	**Res B**	**Peptide A**	**Peptide B**	**Exp. 1**	**Exp. 2**	**Model 1**	**Model 2**	**Distance open**	
VH	54	CH3	417	SKSINSATHYAESVK	LTVDKSR	✖		18.72	44.56	91.84	
VH	78	CH3	417	DDSKSAVYLQMTDLR	LTVDKSR	✖		8.46	54.81	79.32	
CH1	136	CH2	329	GPSVFPLAPSSKSTSGGTAALGCLVK	VSNKALPAPIEK	✖	✖	25.75	21.54	32.45	
CH1	136	CH3	343	GPSVFPLAPSSKSTSGGTAALGCLVK	AKGQPR	✖		23.44	40.7	57.09	
CH2	329	CL	145	VSNKALPAPIEK	EAKVQWK		✖	43.46	17.73	49.57	
VL	1	CH2	341	DILLTQSPAILSVSPGER	TISKAK		✖	65.54	15.64	98.14	
VL	1	CH3	417	DILLTQSPAILSVSPGER	LTVDKSR		✖	41.91	23.29	111.8	

The first six columns identify the domain, residue and tryptic peptides of the identified crosslink. The following two columns show whether the given crosslink was observed in Experiment 1 and/or Experiment 2. The last columns show the measured distance between the Cα atoms of the linking residues in the two closed models and in the open conformation. VH: Variable heavy, VL: Variable light, CH: Constant heavy, CL: Constant light.\

One validating crosslink between (variable heavy chain) Lys43 and (variable heavy chain) Lys54 is intra-domain but with a Cα atom distance in the open conformation of 32.57Å. This is above the commonly accepted “reach” of the BS3 crosslinker (<30Å). However, this crosslink was only observed in Experiment 2, where the distance between these residues in the corresponding Model 2 was reduced, indicating a degree of flexibility in this area.

In order to obtain models of the closed conformation, we used the Modeller harmonic upper bound restraint with threshold at 26 Å and 0.1 standard deviation between the Cα atoms of the residues identified in the crosslinking experiments [21]. Since each crosslink can be either internal i.e. in between residues of the same chain or external i.e. in between residues not belonging to the same chain, we built different models for each possible combination, for an overall total of 16 different models. These models were then relaxed (side chains only) using the FoldX RepairPDB function with default parameters. The final two models, satisfying the observed crosslinks were selected as the ones with the lowest FoldX energy. The following crosslinks (Table 1) are enforced and satisfied for model 1 (Fig 6): internally in each heavy chain between residues 417 and 54, and residue 417 and 78, and externally between residues 136 and 343, and 146 and 329 of different heavy chains, and for model 2 (Fig 6): externally between residues 329 (CH2) and 145 (constant light), residue 1 (variable light) and residue 341 (CH2), residue 1 (variable light) and residue 417 (CH3), and residues 136 and 329 of different heavy chains. Finally, we further optimized the models by relaxing them through the Gromacs MD engine using the ff99SB-ILDN force field. All crosslinks and crosslinked residues in the final models are solvent-accessible and satisfy the BS3 distance range (Fig 6).

A comparison of the two models representing closed conformations is also shown in Fig 6. The overall manner of bending the Fab towards the Fc holds for both models of the closed conformation. There are, however, differences in rotation and bending of the variable light/variable heavy and constant light/CH1 domains, which were suggested by the different sets of cross-linking data. Most likely, the differences in crosslinks observed are due to small differences in experimental conditions and to the semi-quantitative nature of LC-MS. We hypothesize that the IgG molecule *in vitro* rather than having a fixed closed conformation switches between different closed conformations, which supports our suggested mechanism for shielding the Fc region by the Fabs to control the effector functions. Although not proven by our experiments, the superimposed models suggest that the positions/motions of the two Fab arms may show some degree of co-operativity.

### Chemical surface labeling confirms a closed conformation of IgG

Experiments with BS3 crosslinking and biotinylation of native and heat-treated IgG confirmed the “closed” structure of native IgG. Both IFX and IgG subjected to cross-linking with BS3 lost the ability to bind RFs when coated on polystyrene surfaces of ELISA wells, indicating a “locked” conformation (S14a and S14b Fig). In contrast, biotinylation of native IgG did not abolish the ability to bind RFs upon immobilization on a hydrophobic surface, while biotinylation of heat-treated IgG (57 °C) reduced RF binding somewhat, indicating a more accessible Fc in the heat-treated IgG (S14c Fig). This showed that the homobifunctional crosslinker BS3 “locked” IFX and IgG in a conformation unable to react with RFs even if coated on a hydrophobic surface, whereas the biotinylation reagent of approximately the same size as BS3 did not “lock” the IgG.

## Discussion

### Immunoglobulin structure and rheumatoid factors

Igs (antibodies) are extremely important immune defense molecules with a multitude of effector functions. The structure of Igs and their mode of production in response to infections appear to be tailored to immune defense reactions. Igs have a basic structure with two antigen-binding sites and are initially produced as cell surface IgM, but upon antigen stimulation, the producing B cells start a process of affinity maturation, class switching and differentiation, which gives rise to antibody-secreting plasma cells, initially of IgM type but later as IgG, IgA or IgE type, depending on the stimulating antigens/microorganisms and the site of infection [1,24–28]. The structures of Igs have historically been depicted as Y-shaped molecules with two antigen-binding “arms” (Fabs) and an effector part (Fc) (Fig 1). The effector functions are intimately linked to the Fc part and include complement activation and Fc receptor (FcR) interactions [1,2,29–32].

**Fig 7:**
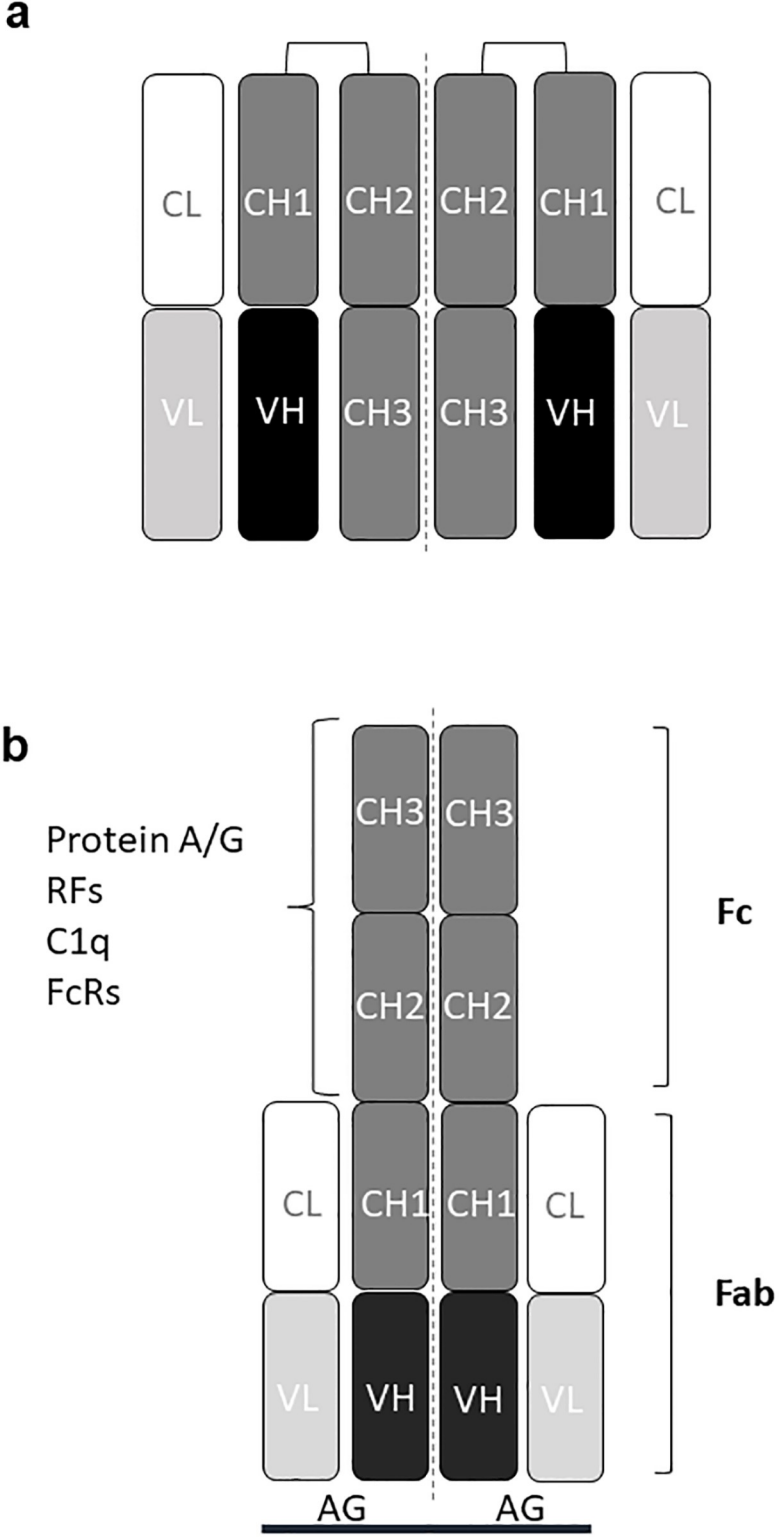
Immunoglobulin G models. (**a**). Graphical model of native (closed) IgG molecule consisting of two heavy chains of γ type and two light chains of either κ or λ type linked by disulphide bridges. The variable part of a heavy chain domain together with the variable part of a light chain domain (VL) together form the antigen binding site. A light chain together with variable heavy and CH1 domains form a Fab (fragment antigen-binding) part (“arm”), and the CH2 and CH3 domains constitute the Fc (fragment constant or crystallizable) part with effector functions. RF binding sites and effector sites for C1/C1q and FcRs reside in the Fc (CH2-CH3) domains and are shielded by the Fab “arms”. (**b**). Model of the conformational change in IgG from closed to open upon antigen binding. Epitopes for RFs, C1q, FcRs and protein A/G are indicated on the Fc. CL: constant light, VL: variable light, VH: variable heavy, CH: constant heavy, Ag: antigen, RF: rheumatoid factor, FcR: Fc receptor.

In patients with autoimmune connective tissue diseases and in some apparently healthy persons, RFs are present in low-high titers together with IgG [5–8]. Also, in blood, Igs circulate in parallel with ample amounts of C1, the first component of the classical complement pathway, and immune cells with cell surface FcRs [1]. Evidently, RFs, C1/C1q and cellular FcRs do not interact strongly with Igs in blood and this would seem to represent a logical paradox. Here, we have shown that while the classical depiction of the Ig(G) structure represents an “open” conformation, the native Ig(G) structure is in fact a “closed” form, with the heavy chains folded back on themselves (Figs 6 and 7). This finding not only resolves the apparent paradox mentioned above, it also illustrates the simple and ingenious natural design of Igs/antibodies.

Thus, the solution to the above paradox is that RFs recognize cryptic epitopes in IgG, which are exposed upon antigen recognition. Previous interpretations of assays for RFs have not recognised that a conformational change in IgG is required for RFs to bind. Rather, in some assays it has been assumed that aggregation of IgG was a prerequisite for RF binding (i.e. an avidity-based hypothesis), neglecting that this cannot account for RF interactions with solid phase-adsorbed IgG. The logical conclusion is that heat-aggregation of IgG is not the mechanism of RF epitope generation, rather the heat-aggregated IgG precipitates (at least for the majority and particularly for the larger complexes). This is presumably also the reason for using short incubation times at 63 °C for generation of RF-reactive epitopes. This incubation leaves enough open, non-aggregated IgG, which can react with RFs in nephelometry and in agglutination assays.

Our data are consistent with each other and illustrate the conclusions in several ways. The solid-phase immunoassays indicate that a conformational change is necessary for RF binding and that the change can occur by covalent immobilization, by physical adsorption on a hydrophobic surface or by simply elevating the temperature to around 50–55 °C. Both of these processes are non-physiological and only serve to illustrate that it is possible to “open” IgG and that the native IgG conformation is different from the RF-interacting form. The physiological correlate is that antibody binding to antigen, e.g on a pathogen surface (c.f. pathogen-associated molecular patterns (PAMPs)), induces a straining force, which releases the Fc part from the Fab “arms”. This would be expected to be most effective, if both antigen-binding sites interact simultaneously with the cognate antigen on the surface of a microorganism (or on a polystyrene surface as used here to mimic a (PAMP) surface). In fact, this process must somehow resemble the release of the Fc part by a “spring” mechanism. The conclusion reached by the simple immunoassay results was substantiated by numerous supporting and inhibition experiments and studies with monoclonal antibodies, in particular the therapeutic monoclonal antibody, IFX. Moreover, the native structure of this antibody was demonstrated to be a closed form by chemical cross-linking experiments in combination with mass spectrometry. From two experiments, seven crosslinks were obtained that did not fit with the open model. In particular two crosslinks from the first experiment linked the variable heavy domain to the CH3 domain, and two crosslinks from the second experiment that bound the N-terminus of the light chain to lysines in CH2 and CH3 showed that IFX had to be tightly folded in order for the residues to be in close proximity. The resulting models (Fig 6) clearly show why RFs, C1/C1q and at least some FcRs do not interact with IgG/IFX, as the Fc CH2 and CH3 domains are for a large part shielded by the Fabs. The two models differ in the way that the Fab folds along the Fc, as the Fab in model 2 is twisted along its own axis relative to the Fab in model 1, but otherwise shields approximately the same region of Fc.

A key to the current results is the use of Ig preparations purified by mild procedures, i.e. IEX chromatography. The IgG preparation used here (IVIG) was produced by IEX chromatography of Igs from volunteer donor plasma [19], and the therapeutic monoclonal antibody, IFX, must also be produced by mild procedures, otherwise, the IVIG and IFX (and also other therapeutic antibodies) would give rise to unwanted adverse reactions upon infusion in recipients (e.g. complement activation) and they would disappear too rapidly from the blood by virtue of FcR and other interactions. In fact, this affords an explanation, why the current observation has gone unnoticed for decades despite the importance of antibodies and the numerous studies of them. Most structural studies of antibodies have been done with protein A-purified antibodies, and many studies with antibody functions and effects have been done with preparations obtained in this way. The discovery of protein A from *Staphylococcus aureus* was a major advance in biotechnology and pathogen biology. Practically, it provided an effective method of antibody affinity purification [33]. Physiologically, it illustrated one of the mechanisms of *S*. *aureus* (and many other pathogens) immune evasion, since the protein A can hijack host Igs and not only use them for “immune camouflage” but at the same time also neutralize antibody effector functions [34]. The current results present a new angle on this perspective; protein A (and protein G) domains have been shown to interact with IgG at the Fc [35–37]. Presumably, protein A with several Ig/Fc-binding domains [38] can somehow “open” the IgGs they bind. When IgGs are subsequently eluted from protein A in the laboratory using low pH, they are obtained in an open conformation for a large part, allowing numerous applications but hampering structural studies by e.g NMR or X-ray crystallography. Similarly, antibodies adsorbed on a surface for e.g. electron microscopy or atomic force microscopy, inevitably obtain an open conformation for a large part.

Besides resolving the apparent RF/C1q/FcR-paradox mentioned above, our results agree with and reconcile several observations in the scientific literature besides the ones described above, and the structural model presented provides a rational explanation for the initiation of effector functions of IgGs (e.g. complement activation and FcR binding) upon antigen binding (Figs 1, 6 and 7).

C1q interacts with IgG Fc through its globular domain, and optimal interaction of C1q with IgG requires binding of IgG to a (pathogen) surface [39,40]. Binding of specific IgGs to their epitopes on a PAMP surface and concomitant opening of the IgG and release of the Fcs would appear to represent an ideal mechanism for presenting C1q epitopes in an optimal configuration. FcRs interact with IgG Fc at the CH2 in a manner involving the structural carbohydrate, and efficient induction of FcR signalling requires bivalent interaction with both sides of the Fc [18,41]. Optimal presentation of symmetric FcR epitopes by open IgG (Fig 7) appears to constitute a logical explanation for activation of FcR signalling by specific IgGs bound to a PAMP surface. In addition to the major closed-open conformational change, minor structural changes in Fab and Fc may occur, simply as a result of a change in straining/pulling forces exerted by the Fab and Fc on each other.

### Immunoglobulin synthesis and B cell biology

The model elaborated on the basis of the present results may well have wider implications. Antibodies are synthesised in a way, where the heavy chain is initially paired with a “surrogate” light chain to be exchanged later with either a κ or λ light chain upon B cell activation/differentiation [1,42]. This process would appear to be fully compatible with our results, demonstrating heavy chain “self-folding”. Secondly, in several species of camelids, fully functional single heavy-chain antibodies, which exhibit high affinity antigen binding, are found in blood [43]. These antibodies must exist in a closed form similar to the one described here, in order to conform with the process of (mammalian) antibody synthesis and secretion and in order to circulate in parallel with complement in blood. Third, several observations indicate that antibody heavy-chain domains may have an indirect influence on antibody affinity [44].

The results also open new avenues for research in antibody and B cell biology. Current models of B cell activation are based on B cell receptor (BCR) dimerization/clustering upon antigen binding [45–47]. Our results are not incompatible with such models, but do suggest a new and simple way of initiating such processes, eventually leading to B cell signalling and activation/differentiation.

Antibodies are synthesized by B cells, which carry cell surface-bound Igs (BCRs) of IgM and IgD type. When these BCRs bind an antigen with a certain threshold affinity they cluster, which leads to antibody-antigen internalisation, breakdown of antigen and presentation of peptide fragments on MHC II. Provided the antigen in question has also been internalised by dendritic cells, which have stimulated T cells, the B cell can receive T cell help and begin to differentiate. This starts a process of antibody affinity maturation and antibody class switching [1,24–28]. In the early phase of the immune response, B cells with sufficient affinity of the cell surface IgM may differentiate to plasma cells secreting pentameric soluble IgM with low affinity but high avidity (effective apparent affinity) [1,2,27,28]. Later in the immune response, these high avidity IgM-producing clones will be outcompeted by B cells producing antibodies of higher affinity and of other classes (primarily IgG (or IgA at epithelial surfaces)) [1,4,24–28], which have an advantage in the competition for antigen (and thereby for T cell help), but it is not entirely clear how the antibody isotype affects clone selection and survival (except that it must relate to antigen competition). At the genomic level, class switching is known to be affected by pattern-recognition receptors and by co-stimulatory molecules and cytokines, which bind to receptors and stimulate intracellular signalling and production of transcription factors, stimulating class switching at certain promoters [1,5,26,28]. However, it is not known either, how this affects clone selection and stimulation. Different types of antibodies have Fc parts with different effector functions and different affinities for receptors on dendritic cells, macrophages and other cells, which recognize the Fc part of the antibodies (Fig 1) [1,2,29–32].

Here, we have shown that the native three-dimensional structure of Igs is adapted to this process by shielding of the Fc by the Fabs. This has the effect that the shielded Fc part of antibodies will only become accessible to FcRs upon binding to antigens on pathogen surfaces. Similarly, the binding site of Igs for C1/C1q appear to be exposed/revealed upon antigen binding, explaining how C1/C1q and Igs can circulate in parallel in blood without significant interactions.

### Autoimmune connective tissue diseases and immunoglobulin structure

In relation to autoimmune connective tissue-disease etiology, the results indicate that the autoantigen for RF formation, most characteristic of rheumatoid arthritis (RA), may be open antibodies/heavy chains released from dead apoptotic/necrotic B cells.

Many questions remain to be investigated and answered in pursuit of the current model. To which degree is the opening reversible? How can the model explain antibody aggregation and denaturation? Can it explain the structural differences among IgG1-4 and among other Ig classes? Can it be used for diagnostic or therapeutic purposes?

Our results were mainly obtained with IgG, but we suggest that it will apply to other antibody classes and perhaps also to antibodies of other animal phyla, which do not conform to the mammalian Ig organization [48]. Actually, some results in the literature support this prediction. Solution studies of IgA have indicated that the Fab arms occupy a position different from the classical one [49], and the mere structure of IgM and its efficient complement-activating property upon multivalent antigen binding is perfectly compatible with our model [50]. Also, cryo electron tomography, although performed on protein A-purified IgG, has indicated that some molecules in solution adopt a more compact conformation compared to the open Y structure [51].

In conclusion, the results presented here show that current Ig(G) models represent an open, Y-shaped conformation (Fig 1) and that native Ig(G) can be depicted by a graphical model as shown in Fig 7, i.e. an m-shaped conformation.

## Materials and methods

### Patient sera

Human sera were from the Biobank at Statens Serum Institut and were used anonymously and in accordance with relevant guidelines and regulations. RF IgM-positive sera had contents from 21–358 IU/mL (average 129 IU/mL), RF IgA-positive sera had values of 30–100 U/mL (average 82 U/mL). In some experiments random pools of 10 RF-positive sera were used. The study was approved by the national committee on health research ethics, Copenhagen, Denmark (Project ID:19980024 PMC and H-15009640) and all experiments were carried out in accordance with relevant guidelines and regulations. Dependent on the experiment conducted, varying numbers of patient sera were used, as noted in the figure legends. The authors did not have direct contact with any patients or donors and were neither involved in drawing nor collection of samples.

### Immunoassays

The different immunoassays used in this work are illustrated in Fig 1 and were carried out essentially as described previously [5,52–54].

For bead-based fluorescent capture immunoassay, human IgGs were immobilised on fluorescent microsphere beads (Luminex, Millipore, Billerica, MA, USA) following the manufacturer’s instructions. Briefly, IgG/IgG1/IgG2/IgG3/IgG4//IgM/IgA (0.06 mg/mL) was coupled to 6.25x10^5^ carboxylated microsphere beads pre-activated with EDC (1-ethyl-3-(3-dimethylaminopropyl)carbodiimide) and N-hydroxysulfosuccinimide using methanesulfonic acid (MES) buffer (50 mM, pH 5.0) and mixing for 2 h at room temperature (RT). Following activation and coupling, the beads were washed and stored in storage buffer (PBS, 0.1% BSA, 0.02% Tween-20, 0.05% NaN_3_, pH 7.4) at 4 °C. Antibody interactions were studied by incubating approximately 5000 beads with human sera (1:100 dilution) for 45 min at RT with or without prior treatments and addition of inhibitors or antigens as indicated. Following incubation, the microsphere beads were washed with assay buffer (PBS, 1% BSA, pH 7.4) (3x1 min). Next, PE-conjugated goat anti-human IgM/IgA/IgG, rabbit anti-human IgG or streptavidin was added to the microsphere beads (4 μg/mL) and incubated for 35 min at RT followed by washing with assay buffer. Finally, approximately 50 beads of each sample were measured on a Bioplex reader (BioSource, Camarillo, CA, USA).

For ELISAs, human IgM/IgA/IgG and other proteins (1 μg/ml in 50 mM sodium carbonate, pH 9.6) were coated with 100 μl/well in Maxisorp polystyrene plates at 5 °C overnight (ON). After blocking at RT with 200 μl/well of TTN buffer (50 mM Tris, pH 7.5, 1% Tween 20, 0.3 M NaCl) and washing (3 x 1 min) with TTN buffer, plates were incubated for 1 h at RT with standards, controls and sera diluted 1:100 in TTN buffer (with or without prior treatments and addition of inhibitors or antigens as indicated). Plates were washed (3 x 1 min) with TTN buffer and incubated 1 h at RT with alkaline phosphatase (AP)-conjugated goat anti-human IgM/IgA/IgG (1:2000) or streptavidin (1:1000) in TTN buffer. After 3 x 1 min washing with TTN buffer, wells were incubated with 100 μl/well *para*-nitrophenylphosphate (pNPP, 1 mg/mL) in AP buffer (1 M ethanolamine, 0.5 mM MgCl_2_, pH 9.8). The absorbance was measured at 405 nm with background subtraction at 650 nm.

For routine RF ELISA, human IgG (10 μg/ml in 50 mM sodium carbonate, pH 9.6) was coated with 100 μl/well in Maxisorp polystyrene plates at 5 °C overnight (ON). After blocking at 5 °C ON with 200 μl/well of buffer A (0.5 M NaCl, 1.5 mM KH_2_PO_4_, 2.7 mM KCl, 8.1 mM Na_2_HPO_4_, 1% BSA, 0.1% Triton X-100, 0.001% phenol red, pH 7.2) and washing (3 x 1 min) with buffer B (0.5 M NaCl, 2.7 mM KCl, 8.1 mM Na_2_HPO_4_, 0.1% Tween 20, pH 7.2) plates incubated 1 h at RT with standards, controls and sera diluted 1:100 in buffer A. Plates were washed (3 x 1 min) with buffer B and incubated 1 h at RT with horseradish peroxidase-conjugated rabbit IgG to human IgM or IgA diluted 1:9000 or 1:5000, respectively, in buffer B. After 3 x 1 min washing with buffer B, wells were incubated for 15 min with 50 μl/well 0.03% (w:w) H_2_O_2_, 0.7 mg/ml o*rtho*-phenylenediamine substrate in buffer C (67 mM Na_2_HPO_4_, 35 mM citric acid, pH 5.0), and the reaction was then stopped by addition of 150 μl/well 1 M H_2_SO_4_. The absorbance was measured at 492 nm with background subtraction at 690 nm. The IgM RF concentration is given as IU/ml and IgA RF concentration is given as U/ml. The cut-off value of the RF analyses (20 IU/ml for IgM RF, 20 U/ml for IgA RF) is set to compensate for the fact that many apparently healthy persons (especially with older age) contain RFs (values between 15 and 20 (I)U/ml are considered as a twilight-zone).

### Chemical crosslinking and surface labelling

IFX samples were diluted to a concentration of 0.5 and 1μg/μL in 100mM PBS buffer. The samples were incubated at 37 °C. BS3 crosslinker (ProteoChem, Hurricane, UT) was incubated at room temperature (RT) before use. BS3 was dissolved in water for a final concentration of 22mM BS3. BS3 was added to the antibody samples to a final concentration of 2.2mM. The samples were incubated at RT for 45min. Crosslinking was quenched using 100mM NH_4_HCO_3_ for 15min. The samples were lyophilized prior to SDS-PAGE.

Chemical modification with various reagent was done by diluting IFX or IgG to 1 mg/mL in 50 mM sodium phosphate, pH 9 (PB) and allowing them to react with the indicated concentrations of BS3 (dissolved in dimethylsulfoxide) or biotin-N-hydroxysuccinimidester dissolved in dimethylformamide. After reaction, the Ig was dialysed against phosphate-buffered saline (PBS), pH 7.2.

### SDS-PAGE and immunoblotting

The samples were dissolved in sample buffer (70 mM LDS (lithium dodecylsulfate), 10 mM dithiothreitol, 10% (v:v) glycerol, 0.05 M Tris pH6.8) and incubated at 70°C for 10 min. The samples were loaded onto a 4–12% Bis-Tris gel PAGE gel or a 4–20% Tris PAGE gel along with a protein ladder, PageRuler Plus (Bio-Rad, Hercules, CA). Electrophoresis was carried out for 30-45min at 200 V and 400 mA. The proteins in the gels were stained using a Coomassie Brilliant Blue-based protein stain for 15 min. The gels were destained in ultra high quality (UHQ) water for 30 min, twice.

Gels were electroblotted to polyvinyldifluoride membranes using an iBlot apparatus. Membranes were blocked in TTN buffer and incubated with an RF pool diluted 1:500 in TTN for 1 h. After 3 washes in TTN, membranes were incubated with AP-conjugated GaHIgM, GaHIgA, or GaHIgG diluted 1:2000 in TTN and incubated 1 h. After further 3 washes, bound antibodies were visualized using 5-bromo-4-chloro-3-indolyl-phosphate/nitroblue-tetrazolium (BCIP/NBT) substrate tablets.

### Sample preparation

Relevant gel bands were excised from the gel into separate low-binding polypropylene tubes. In-gel trypsin digestion was performed as described [55]. Each sample was desalted using an in-house made Poros Oligo R2 reverse phase (Applied Biosystems, Foster City, CA, USA) column packed in a Rainin pipette tip.

### LC-MS/MS

The digested peptides were dissolved in 0.1% TFA and approximately 1μg peptides were analyzed using an EASY-nanoLC 1000 system (Thermo Scientific, Germany) coupled to either the Orbitrap Fusion Lumos Tribrid mass spectrometer or the Q-Exactive HF mass spectrometer. The peptides were loaded onto a 2.5 cm in-house packed Reprosil-Pur 120 C18-AQ (5 μm: Dr. Maisch GmbH, Germany) precolumn with an internal diameter of 100μm and eluted directly onto a 19 cm in-house packed Reprosil-Pur C18-AQ column (3 μm; Dr. Maisch GmbH, Germany) with an internal diameter of 75 μm. A 104 min HPLC gradient with a flow rate of 250 nL/min was used, which used an increasing concentration of Solvent B (95% ACN, 0.1% FA) in the following increments: 1–3% for 3 min, 3–25% for 80 min, 25–45% for 10 min, 45–100% for 3 min and 100% for the final 8 min. Solvent A was 0.1% FA.

Both mass spectrometers were operated in data dependent acquisition mode and each analysis started with an MS1 scan performed/detected in the Orbitrap (resolution: 120.000, scan range: 300–2000 m/z, AGC target: 400.000, maximum injection time: 100 ms, RF lens: 30%). For each MS1 spectrum, the 15 most intense peaks were selected for MS2. Monoisotopic Peak determination was set to peptide and dynamic exclusion was switched on (exclude after 1 time, exclusion duration: 15 s, mass tolerance: ±10ppm, exclude isotopes). The selected MS2 precursors were isolated in the quadrupole at 1.0 Th (m/z) and fragmented using HCD at a collision energy of 30%. MS2 fragments were detected in the Orbitrap (resolution: 30.000, first mass: 110 m/z, AGC target: 200.000).

### Data analysis

Raw data files were converted to mgf format by Proteome Discoverer 2.2 (Thermo Fischer, U.S.A). The data was further filtered using the dynamic cutoff standard settings of MassAI (www.massai.dk). The sequence of IFX was acquired from the PDB structure 5VH3, and the human Ig heavy-constant γ chain (SwissProt P01857). The sequence was used for examining BS3 crosslinks by the MassAI search engine with the parameters: 10 ppm MS accuracy, 0.10 Da MS/MS accuracy, trypsin for the enzyme, four allowed missed cleavages, carbamidomethylation of cysteine as fixed modification and oxidation of methionine, BS3-water dead-end and BS3-ammonia dead-end of lysine residues and the protein N-terminus as variable modifications. The cross-linker was selected to be non-labeled (d0) BS3. Searches for cross-links were performed using MassAI and annotated as validating or over-length cross-links (Table 1). Crosslinks were manually validated and crosslinks spectra were only approved if b- and/or y-ions from both crosslinked peptides were present in the spectrum. Representative scans for each cross-link were annotated and are presented in S10 Fig.

### Protein unfolding

Eva Green fluorescence assay of protein unfolding was done as described [18].

### Size exclusion chromatography

Size exclusion chromatography (gel filtration) was done using a Superose 6 column mounted on an ÄKTA FPLC system (GE Healthcare/Pharmacia, Uppsala, Sweden). The column was equilibrated in phosphate-buffered saline (PBS), and the flow rate was 0.5 mL/min. One hundred μL samples (pooled sera) were injected and 1 mL fractions were collected and assayed by ELISA for IgM, IgA, IgG and for IgM and IgA RFs.

### Statistics

Statistical comparison of data sets was done by ANOVA using Prism (Graphpad Software, San Diego, CA, USA). P values < 0.05 were considered significant (*: p < 0.05, **: p < 0.005, ***: p < 0.0005).

## Supporting information

S1 Fig RFs are present in RA serum in parallel with IgG. One hundred μL of pooled RA sera were chromatographed on a Superose 6 size exclusion column using an ÄKTA FPLC system (Phramacia/GE Healthcare, Uppsala, Sweden). The buffer was phosphate-buffered saline (PBS, 50 mM sodium phosphate, pH 7.2, 0.15 M NaCl) and the flow rate was 0.5 mL/min. One mL fractions were collected and tested for IgM, IgA and IgG and for RF IgM and IgA by ELISA. Testing for IgM, IgA and IgG was carried out by coating fractions directly (1:100) in wells of microtitre plates, blocking with TTN buffer, incubating with AP-conjugated GaHIgM/A/G 1:2000 in TTN buffer and developing with pNPP. Testing for RFs was done as described in materials and methods. Stippled lines represent ELISA readings (A405) between 0–0.5 and solid lines represent ELISA readings between 0.5–1. The elution position of molecular weight markers are indicated above the elution profile.
(PDF)

S2 Fig RFs are present in RA serum in parallel with IgG and forms a precipitate with heat-treated IgG. (**a-d**). Photographs of RA (**a,b**) and HD (**c,d**) sample pools after incubation with heat-treated IgG. Note precipitate in **a** and **b** (before and after centrifugation) but not in **c** and **d** (before and after centrifugation). One hundred μL of pooled RA or HD sera were mixed with 10 μL heat-treated IgG (57 °C, overnight, heating cabinet) and incubated 1 h at room temperature and then at 5 °C overnight. This resulted in a white precipitate in the RA pool but not in the HD pool. The precipitate in the RA pool was isolated by centrifugation, washed twice with water and dissolved in 100 μL non-reducing sample buffer. Half of this was mixed with non-reducing sample buffer and half was mixed with reducing sample buffer followed by 3 min boiling. The samples were then loaded in wells of two 4–20% SDS-PAGE gels and subjected to electrophoresis. Half of the gels were stained with Coomassie Brilliant Blue (**e,h**) and half were electroblotted to PVDF membranes. The membranes were used for immunoblotting using AP-conjugated GaHIgM (**f**) or GaHIgA (**i**) with BCIP/NBT dvelopment. After scanning, the membranes were further incubated with AP-conjugated GaHIgG and again developed with BCIP/NBT. Gels and blots were scanned using a GelDoc XR+ Molecular Imager (BioRad, Hercules, CA. USA).
(PDF)

S3 Fig Gelfiltration chromatography of RA and HD sera pools after addition of heat-treated IgG and centrifugation (supernatants from S2 Fig). The gelfiltration and analysis of fractions were done as described in S1 Fig.
(PDF)

S4 Fig RFs IgM in RA sera do not react with native IgG but reacts with heat-treated IgG in a capture ELISA. Wells of a microtitre plate were coated overnight with GaHIgM (1:1000 in carbonate buffer, pH 9.6), washed and blocked with TTN buffer and incubated with native IgG kept at 5 °C or heat-treated IgG (57 °C, over night) (1 mg/mL, 1:1000 in TTN buffer), followed by washing and 1 h incubation with AP-conjugated GaHIgG (1:2000 in TTN buffer). Wells were again washed with TTN buffer and then developed with pNPP. The absorbance was read at 405 nm with background subtraction at 650 nm.
(PDF)

S5 Fig RF reactivity. (**a**). Reaction of RFs (IgM) with ion exchange-purified native (4 °C, 21 °C) or heat-treated IgG (34 °C– 64 °C) when coated on the polystyrene surface of ELISA plate wells. (**b**). Reactivity of RFs (IgM) with native (control) or heat-treated bIgG in a bridging ELISA with IgG (non-heated) coated on the polystyrene surface of ELISA plate wells. (**c, d**). Reactivity of immobilised protein A (**c**) and protein G (**d**) with native and heat-treated (57 °C) bIgG in ELISA. (**e**). Temperature dependency for reaction of heat-treated IgG with immobilised protein G in ELISA. Bound IgG was detected with RaHIgG (**f**). Reaction of immobilised RFs with native (room temperature (RT), 37 °C) and heat-treated (47 °C—67 °C) bIgG. Figures show means of double determinations and are from one experiment out of two.
(PDF)

S6 Fig RFs do not react with native or heat-treated IgA or IgM in a bead-based bridging assay with immobilized IgG. Nine sera each of RF IgA-positive (RF IgA^+^), RF IgA-negative (RF IgA^-^), RF IgM-positive (RF IgM^+^) and RF IgM-negative (RF IgM^-^) sera were tested for bridging of native bIgA (**a**), bIgM (**b**) or heat-treated bIgA (**d**) or bIgM (**e**) to immobilized IgG. Controls (**c, f**): beads had no immobilized IgG. The positive control was rabbit antibodies to human IgG (RaHIgG).
(PDF)

S7 Fig IgG temperature-induced unfolding. The thermal unfolding of IgG was measured by an Eva Green fluorescence assay as described [18]. Human calreticulin, which has a low melting temperature (T_m_) was used as an internal standard. The figure is based on triple determinations (shown in different colors) and is from one representative experiment out of two.
(PDF)

S8 Fig Cross-reactivity of affinity-purified RFs with IgG and IFX. RFs were allowed to bind immobilized IgG (**a**) or IFX (**b**) in ELISA wells, then eluted by lowering the pH and subsequently tested for binding to IgG or IFX, respectively, in the same assay. Figures show means of double determinations and are from one experiment out of two.
(PDF)

S9 Fig RF reactivity. (**a**). Reactivity of RFs (IgM) with native (37 °) or heat-treated IFX (39 ° - 57 °C) in a bridging ELISA with IFX (non-heated) coated on the polystyrene surface of ELISA plate wells. (**b**). Reaction of RF IgM with IgG, different human therapeutic IgGs (IFX, Ipilimumab, Nivolumab), the TNFR-Fc biological drug Enbrel and three murine monoclonal antibodies (Mab) (02–01, 99–01, 273–01). Figures show means of double determinations and are from one representative experiment out of two.
(PDF)

S10 Fig IgG structure. (**a**). Binding of native or heat-treated IgG to Concanavalin A. IgG pre-incubated at the indicated temperatures was incubated in ELISA plate wells coated with protein G and then tested for reaction with ConA (biotinylated, allowing detection with AP-conjugated streptavidin). (**b**). IgG conformational change upon heating allowing interaction with C1q. C1q was immobilized on the surface of ELISA wells and incubated with bIgG, which had been pre-incubated at the indicated temperatures. The interaction was maximal, when bIgG had been subjected to heating at 57 °C. Figures show means of double determinations and are from one representative experiment out of two.
(PDF)

S11 Fig IgG structure and RF reactivity. (**a, b**). SDS-PAGE analysis of IgG subjected to cleavage with the hinge-specific protease Ide S. IgG was preincubated at temperatures from 5 °C to 57 °C and then incubated with or without Ide S. Samples were analysed by SDS-PAGE on 4–20% gels without (**a**) and with (**b**) reduction with DTT. (**c, d**). SDS-PAGE (**a**) and Western immunoblotting (**b**) analysis of intact (lanes 3, 7) and IdeS-cleaved (lanes 5, 9) IgG with a pool of RF-IgM-positive sera. Note that strong reaction is seen with Fc-containing bands (*), while only a faint non-specific reaction is seen with F(ab’)_2_. Also note that the mobility of Fc is influenced by reduction. Figures are from one representative experiment out of two. Bands are assigned as follows;
IgG (Fab_2_Fc)One-hinge-cleaved IgG (FabFc)Fab_2_FcHeavy chainLight chainFc (lower mobility than in lane 4 due to intact, non-reduced S-S bonds)Light chain, Variable heavy-CH1.
(PDF)

S12 Fig In gel trypsin digestion. SDS-PAGE gels of BS3-crosslinked IFX in experiment 1 (0.5 μg/μL IFX) and experiment 2 (1μg/μL IFX), respectively. Both gels show a single band between 130–180 kDa markers, corresponding to monomeric crosslinked IFX molecules. These bands were excised for analysis.
(PDF)

S13 Fig MS/MS spectra of the crosslinked peptides presented in Table 1. In the spectra, y- and b-fragment ions are presented in blue and green, respectively. Purple peaks represent neutral losses, pink peaks represents isotope ions, and red peaks are unassigned ions. Below each spectrum is the annotated sequences of peptide A (top) and peptide B (bottom). Blue and green marks above and below the sequence indicates observed y- and b-ions, respectively. The cross-linked residues are marked in red and their sequence numbers and domains are shown in the top right corner.
(PDF)

S14 Fig Chemical surface labelling and cross-linking of IgG. (**a**). Effect of bis-succinimidyl-suberate (BS3) cross-linking on RF IgM reaction with coated IFX as determined by ELISA. (**b**). Effect of BS3 cross-linking on RF IgM reaction with coated IgG as determined by ELISA. (**c**). Effect of biotinylation on RF IgM reaction with coated IgG/bIgG as determined by ELISA. The experiments were done by using the modified IFX/IgG as antigen in the routine RF IgM ELISA and figures show one representative experiment out of two.
(PDF)

S1 Table Supporting information, raw data files. Raw data for Figs 2–5, S1–S10 and S14 Figs.
(XLSX)
