# Supplementary figures and images for: Immunoglobulin G structure and rheumatoid factor epitopes
Source: PLoS One. 2019 Jun 14;14(6):e0217624. doi: 10.1371/journal.pone.0217624 (PMC6568389; doi:10.1371/journal.pone.0217624)

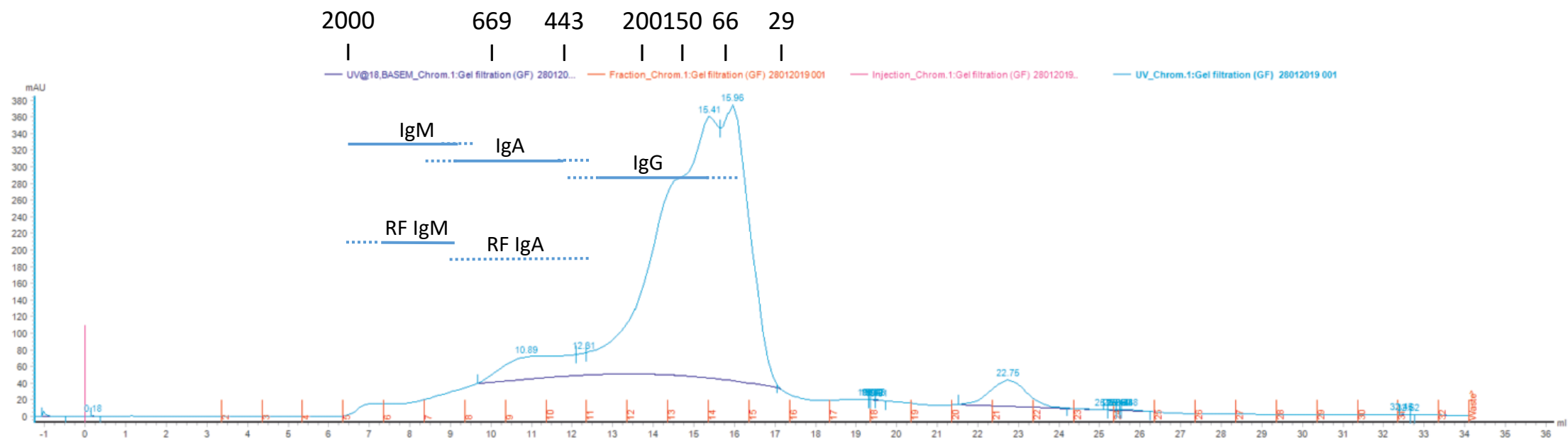

Supplement: S1 Fig — One hundred μL of pooled RA sera were chromatographed on a Superose 6 size exclusion column using an ÄKTA FPLC system (Phramacia/GE Healthcare, Uppsala, Sweden). The buffer was phosphate-buffered saline (PBS, 50 mM sodium phosphate, pH 7.2, 0.15 M NaCl) and the flow rate was 0.5 mL/min. One mL fractions were collected and tested for IgM, IgA and IgG and for RF IgM and IgA by ELISA. Testing for IgM, IgA and IgG was carried out by coating fractions directly (1:100) in wells of microtitre plates, blocking with TTN buffer, incubating with AP-conjugated GaHIgM/A/G 1:2000 in TTN buffer and developing with pNPP. Testing for RFs was done as described in materials and methods. Stippled lines represent ELISA readings (A405) between 0–0.5 and solid lines represent ELISA readings between 0.5–1. The elution position of molecular weight markers are indicated above the elution profile. (PDF) [file pone.0217624.s001.pdf]

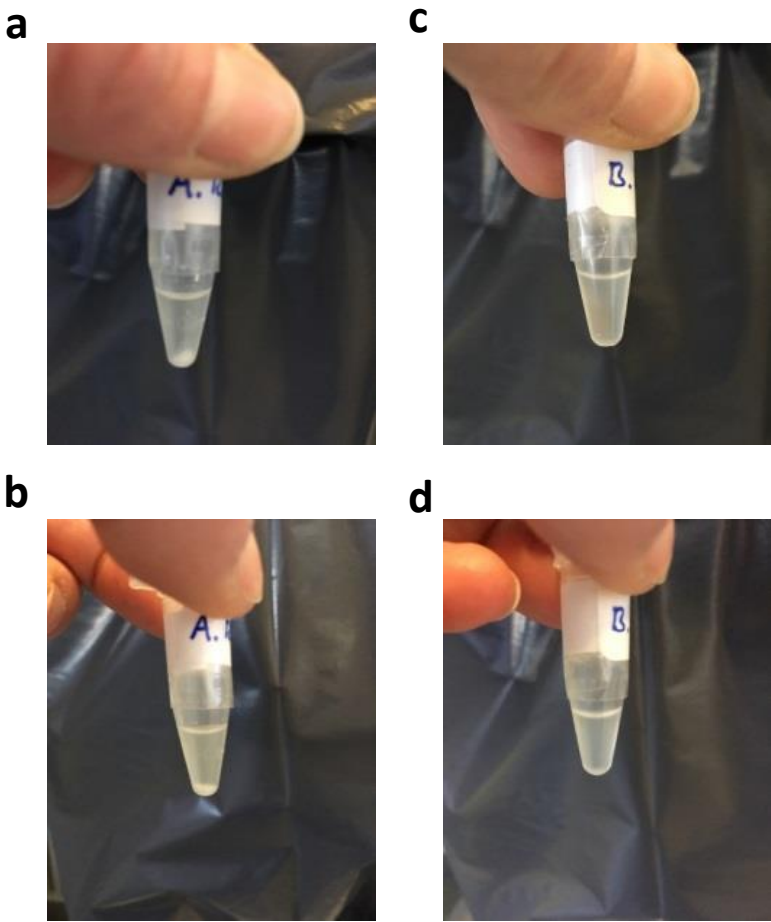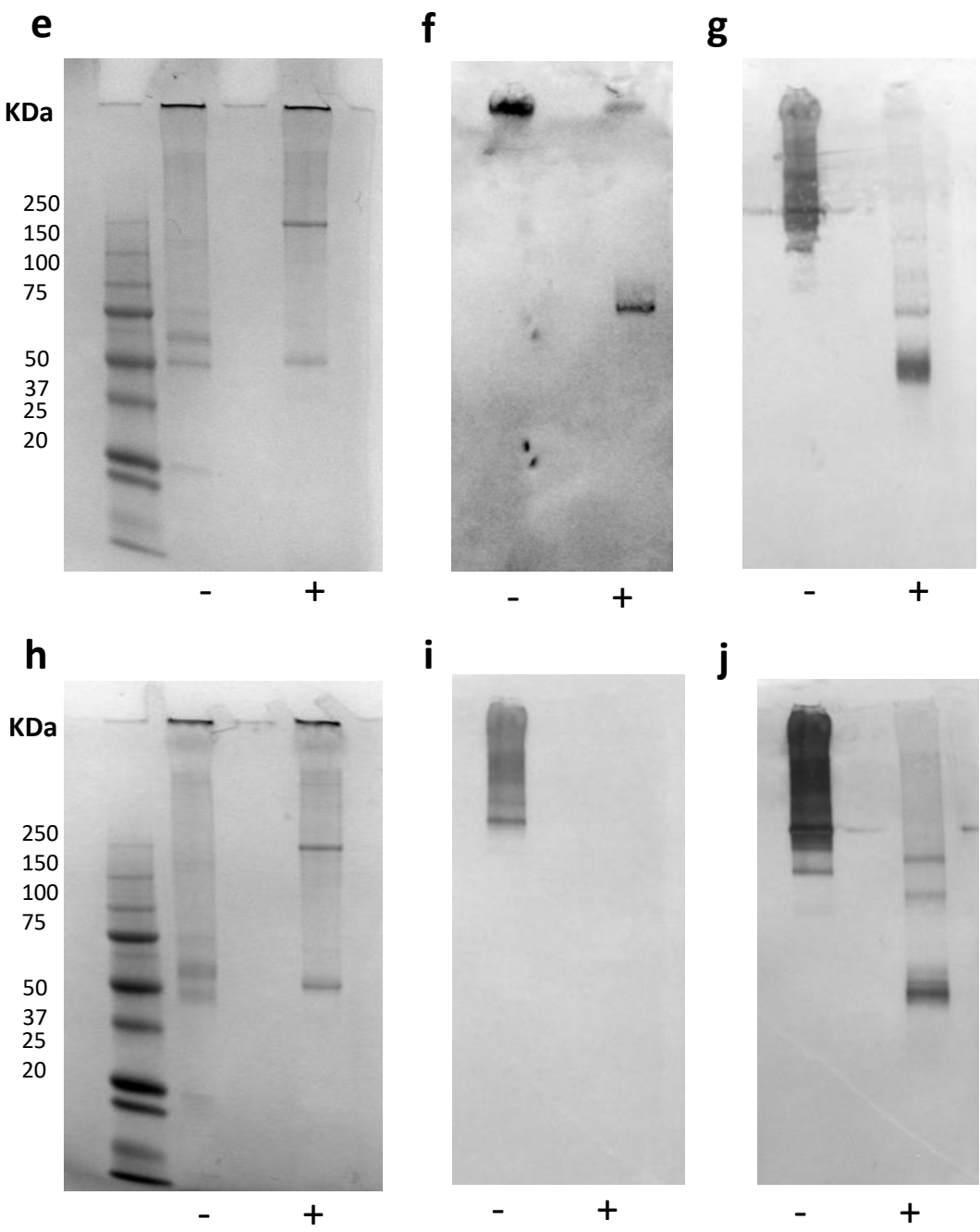

Supplement: S2 Fig — (a-d). Photographs of RA (a,b) and HD (c,d) sample pools after incubation with heat-treated IgG. Note precipitate in a and b (before and after centrifugation) but not in c and d (before and after centrifugation). One hundred μL of pooled RA or HD sera were mixed with 10 μL heat-treated IgG (57 °C, overnight, heating cabinet) and incubated 1 h at room temperature and then at 5 °C overnight. This resulted in a white precipitate in the RA pool but not in the HD pool. The precipitate in the RA pool was isolated by centrifugation, washed twice with water and dissolved in 100 μL non-reducing sample buffer. Half of this was mixed with non-reducing sample buffer and half was mixed with reducing sample buffer followed by 3 min boiling. The samples were then loaded in wells of two 4–20% SDS-PAGE gels and subjected to electrophoresis. Half of the gels were stained with Coomassie Brilliant Blue (e,h) and half were electroblotted to PVDF membranes. The membranes were used for immunoblotting using AP-conjugated GaHIgM (f) or GaHIgA (i) with BCIP/NBT dvelopment. After scanning, the membranes were further incubated with AP-conjugated GaHIgG and again developed with BCIP/NBT. Gels and blots were scanned using a GelDoc XR+ Molecular Imager (BioRad, Hercules, CA. USA). (PDF) [file pone.0217624.s002.pdf]

A

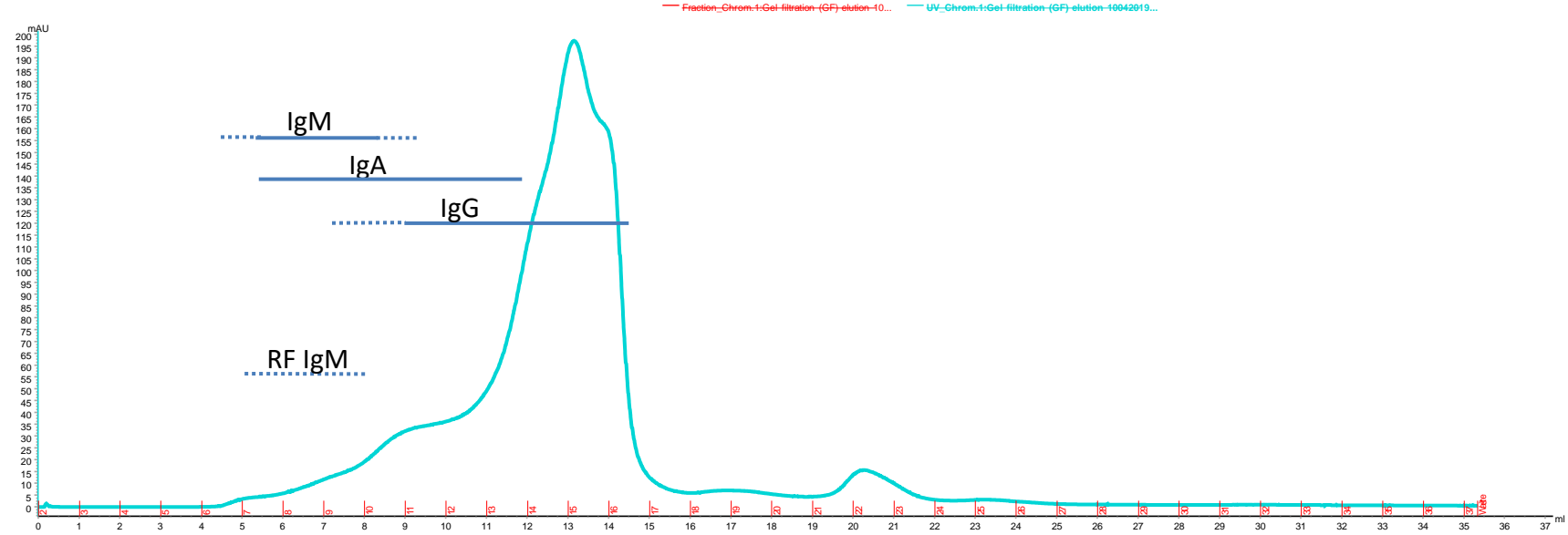

B

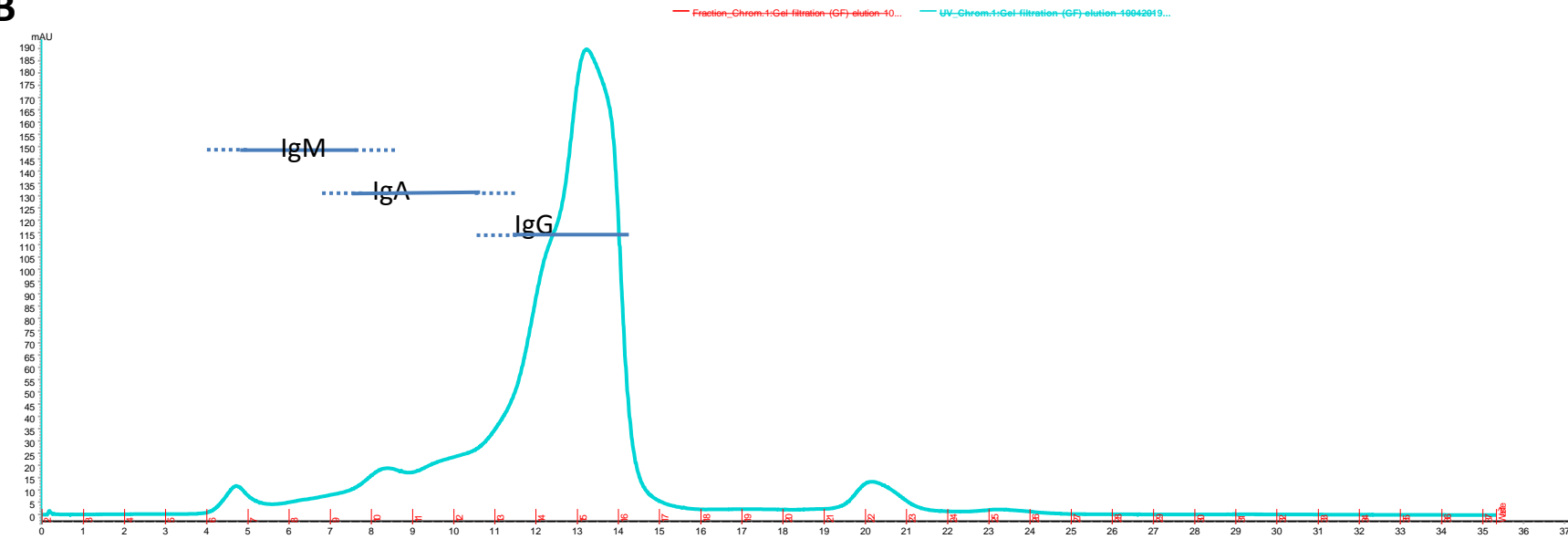

Supplement: S3 Fig — The gelfiltration and analysis of fractions were done as described in S1 Fig. (PDF) [file pone.0217624.s003.pdf]

Fig S4

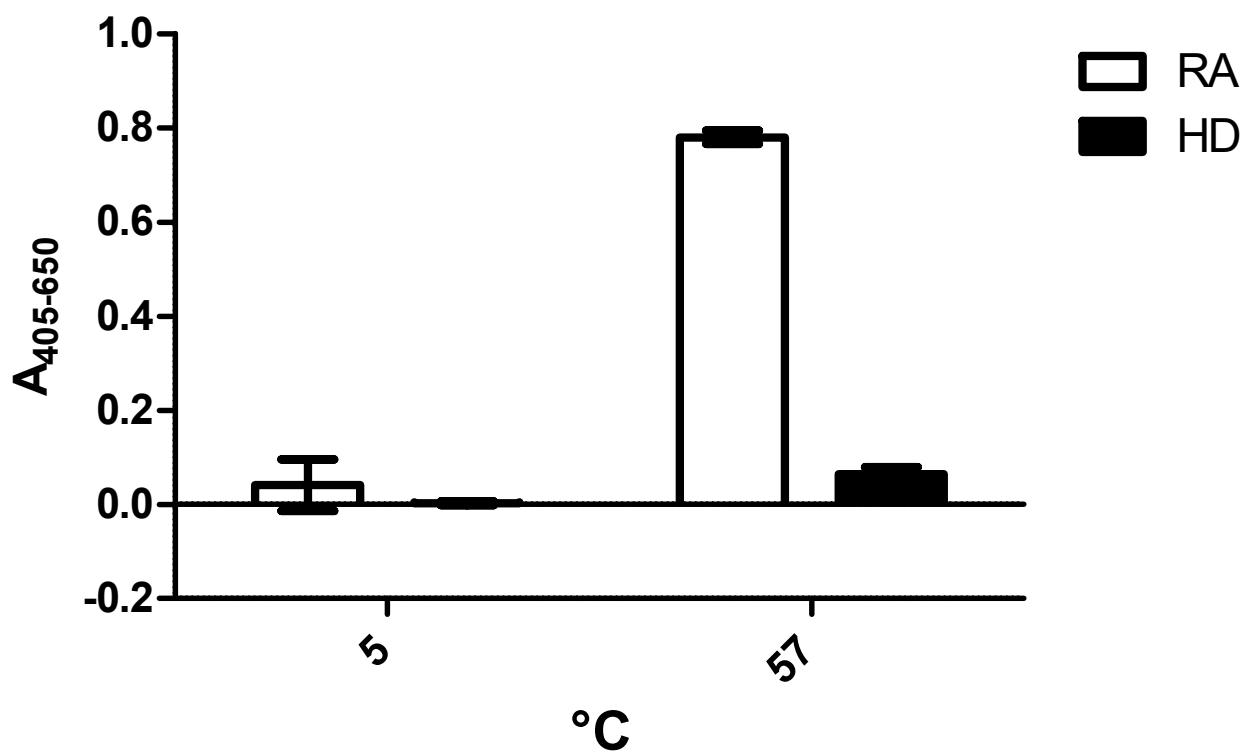

Supplement: S4 Fig — Wells of a microtitre plate were coated overnight with GaHIgM (1:1000 in carbonate buffer, pH 9.6), washed and blocked with TTN buffer and incubated with native IgG kept at 5 °C or heat-treated IgG (57 °C, over night) (1 mg/mL, 1:1000 in TTN buffer), followed by washing and 1 h incubation with AP-conjugated GaHIgG (1:2000 in TTN buffer). Wells were again washed with TTN buffer and then developed with pNPP. The absorbance was read at 405 nm with background subtraction at 650 nm. (PDF) [file pone.0217624.s004.pdf]

Fig S5

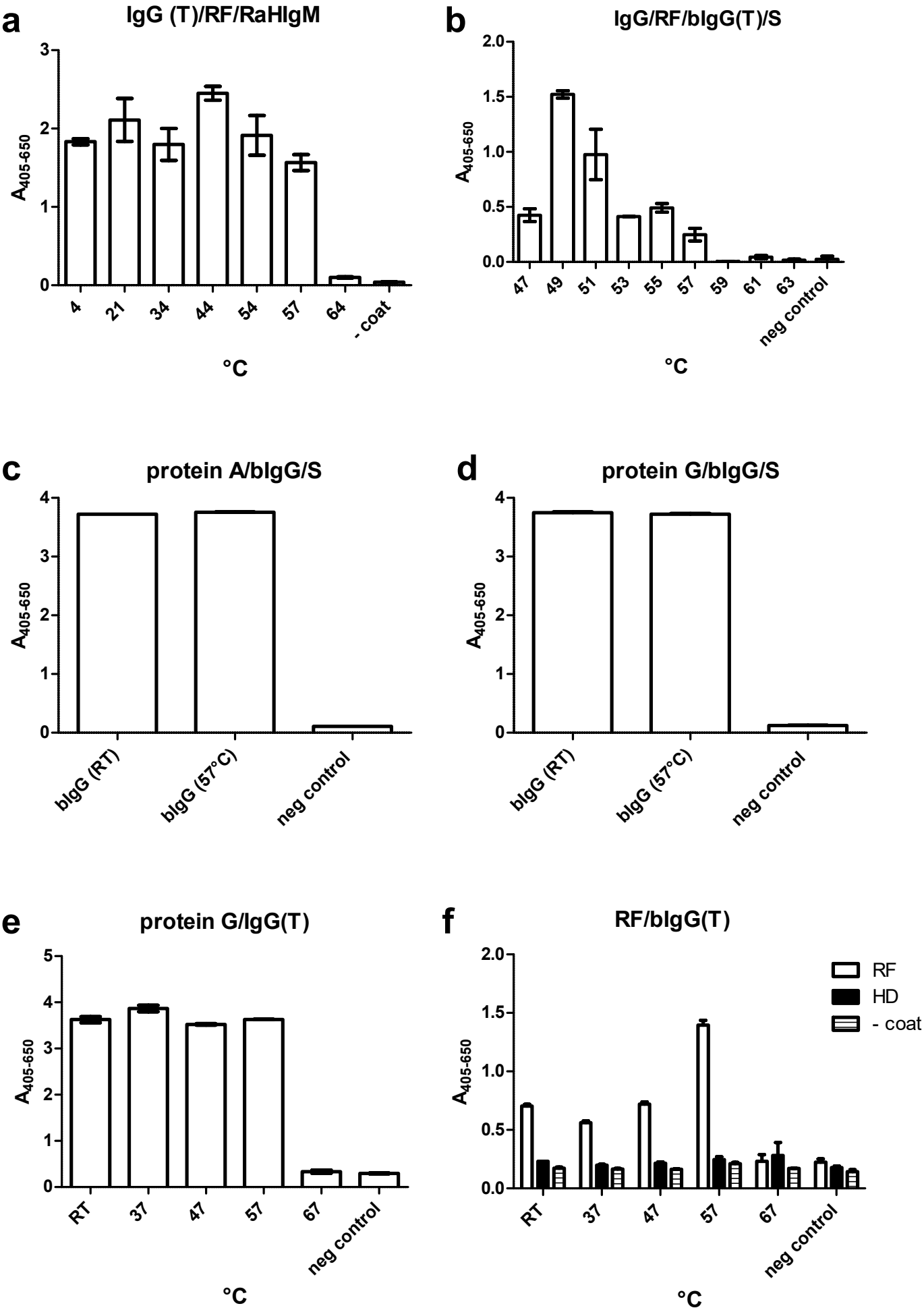

Supplement: S5 Fig — (a). Reaction of RFs (IgM) with ion exchange-purified native (4 °C, 21 °C) or heat-treated IgG (34 °C– 64 °C) when coated on the polystyrene surface of ELISA plate wells. (b). Reactivity of RFs (IgM) with native (control) or heat-treated bIgG in a bridging ELISA with IgG (non-heated) coated on the polystyrene surface of ELISA plate wells. (c, d). Reactivity of immobilised protein A (c) and protein G (d) with native and heat-treated (57 °C) bIgG in ELISA. (e). Temperature dependency for reaction of heat-treated IgG with immobilised protein G in ELISA. Bound IgG was detected with RaHIgG (f). Reaction of immobilised RFs with native (room temperature (RT), 37 °C) and heat-treated (47 °C—67 °C) bIgG. Figures show means of double determinations and are from one experiment out of two. (PDF) [file pone.0217624.s005.pdf]

Fig S6

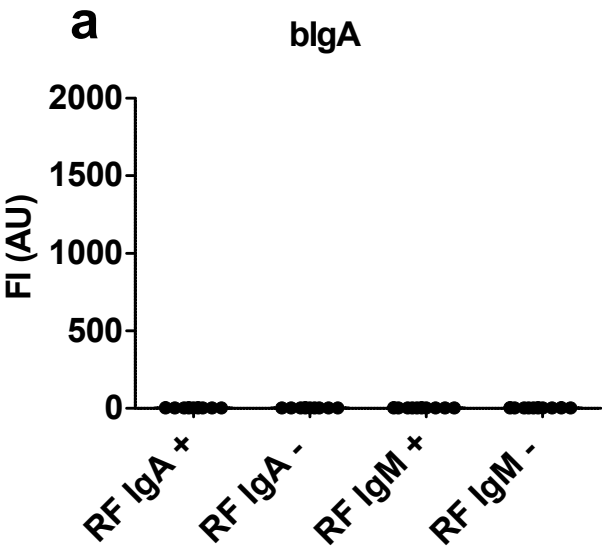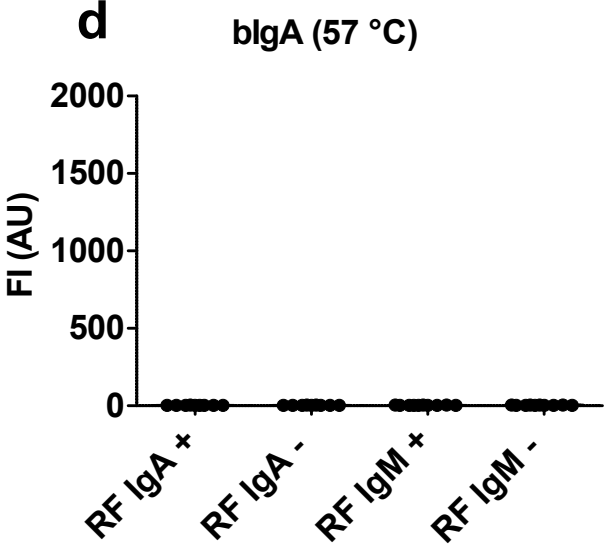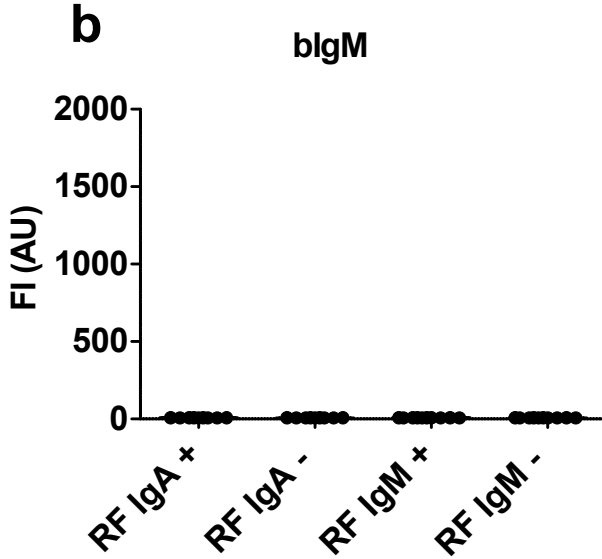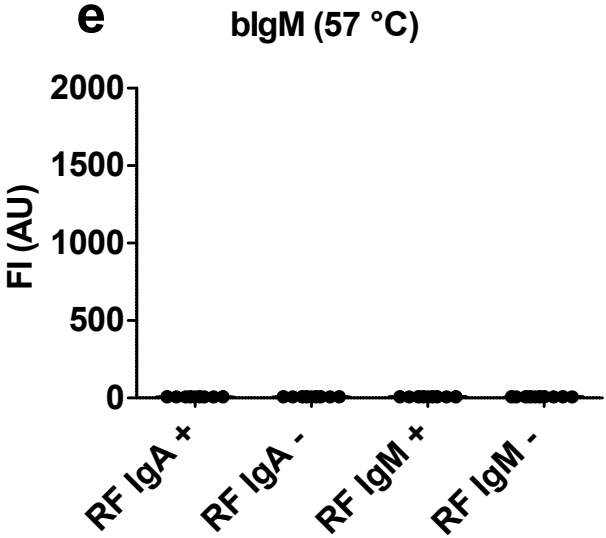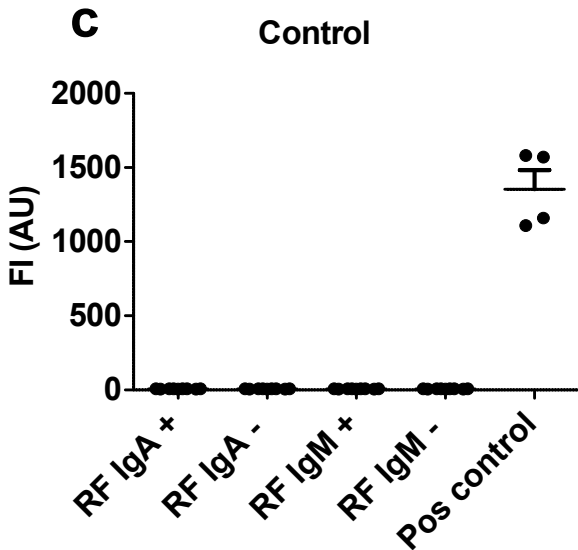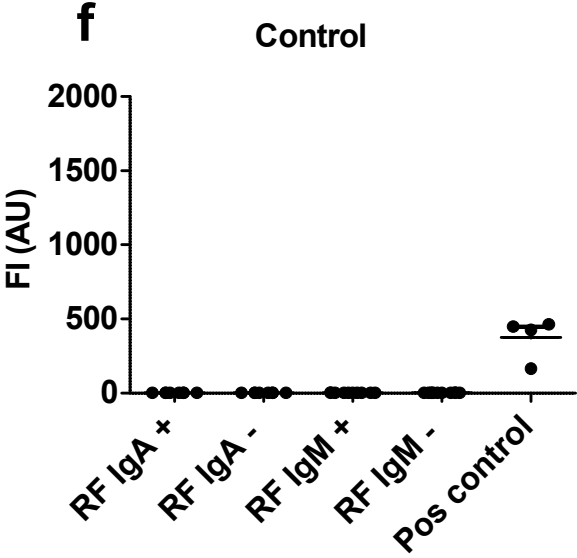

Supplement: S6 Fig — Nine sera each of RF IgA-positive (RF IgA+), RF IgA-negative (RF IgA-), RF IgM-positive (RF IgM+) and RF IgM-negative (RF IgM-) sera were tested for bridging of native bIgA (a), bIgM (b) or heat-treated bIgA (d) or bIgM (e) to immobilized IgG. Controls (c, f): beads had no immobilized IgG. The positive control was rabbit antibodies to human IgG (RaHIgG). (PDF) [file pone.0217624.s006.pdf]

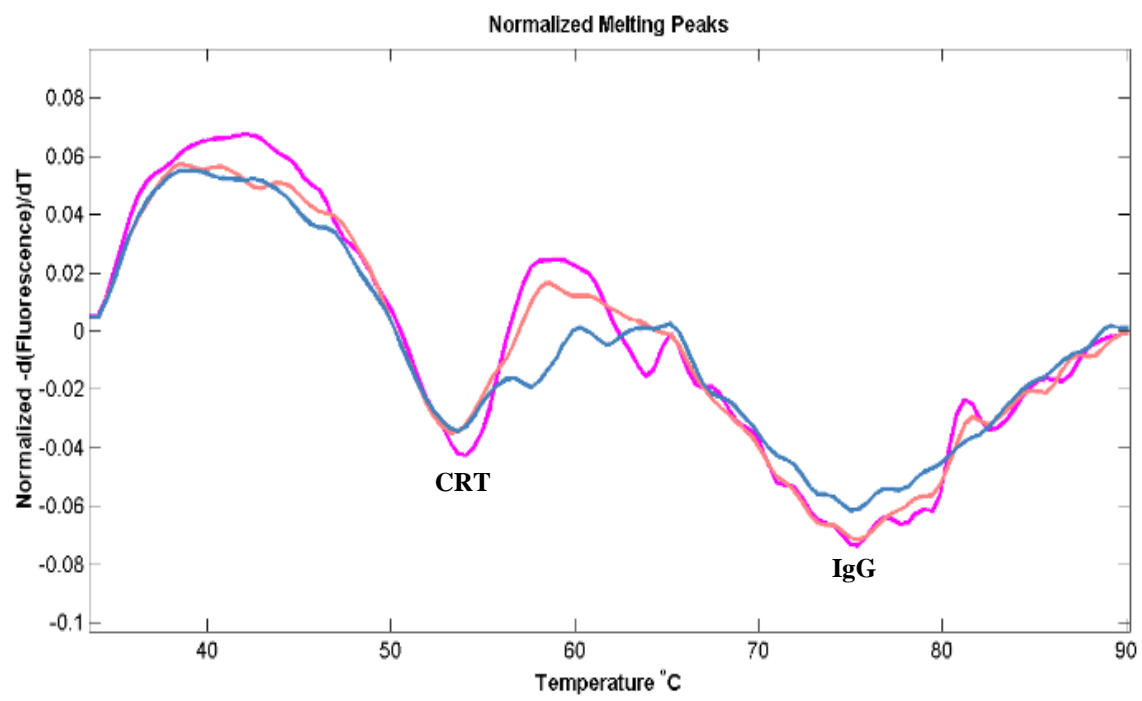

Supplement: S7 Fig — The thermal unfolding of IgG was measured by an Eva Green fluorescence assay as described [18]. Human calreticulin, which has a low melting temperature (Tm) was used as an internal standard. The figure is based on triple determinations (shown in different colors) and is from one representative experiment out of two. (PDF) [file pone.0217624.s007.pdf]

**Fig S8**

**a**

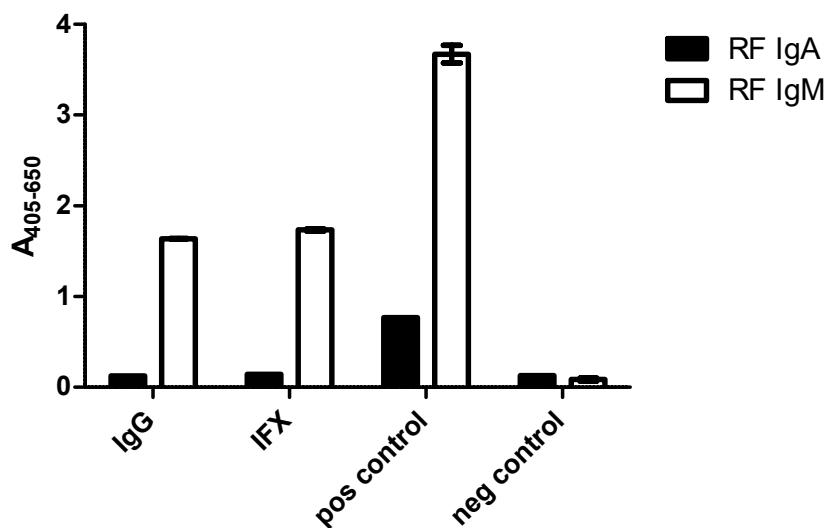

**b**

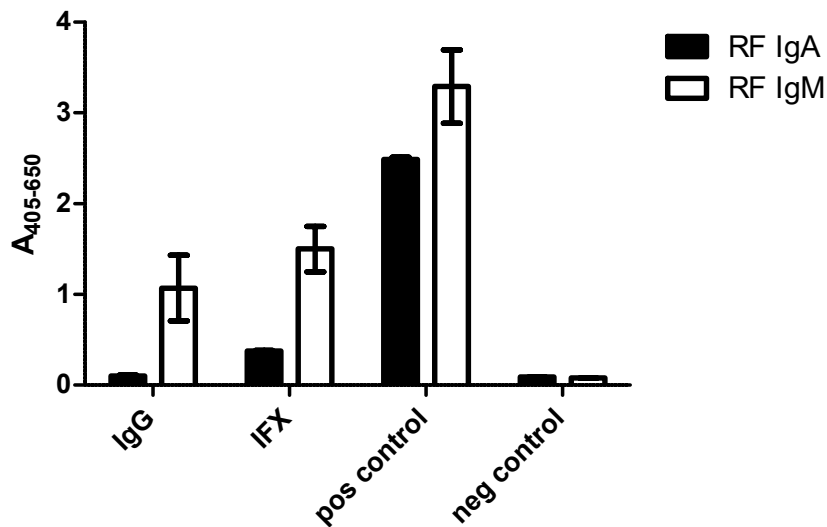

Supplement: S8 Fig — RFs were allowed to bind immobilized IgG (a) or IFX (b) in ELISA wells, then eluted by lowering the pH and subsequently tested for binding to IgG or IFX, respectively, in the same assay. Figures show means of double determinations and are from one experiment out of two. (PDF) [file pone.0217624.s008.pdf]

Fig S9

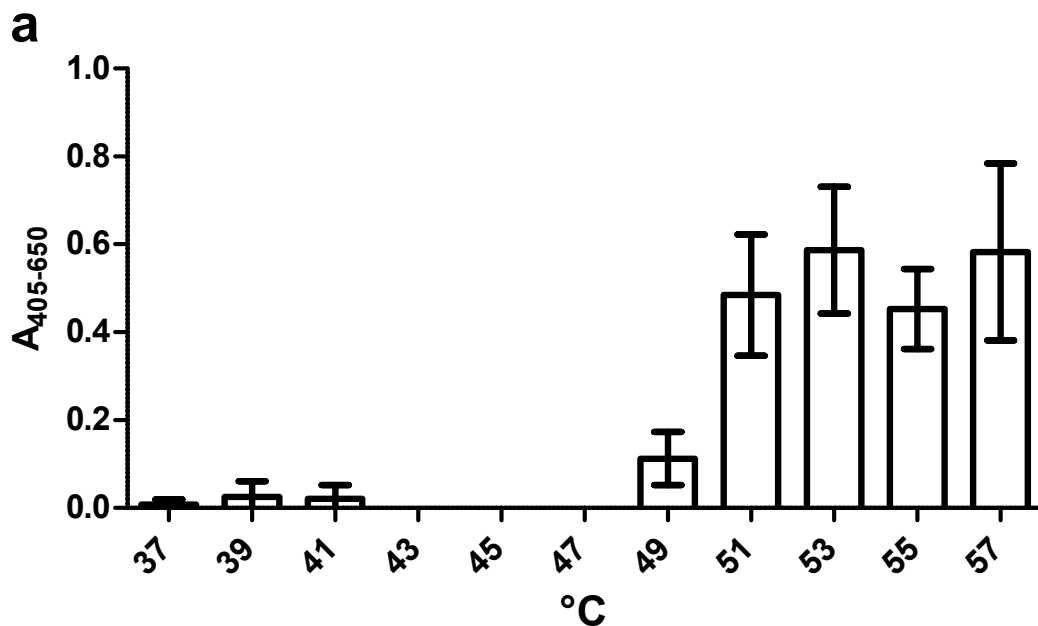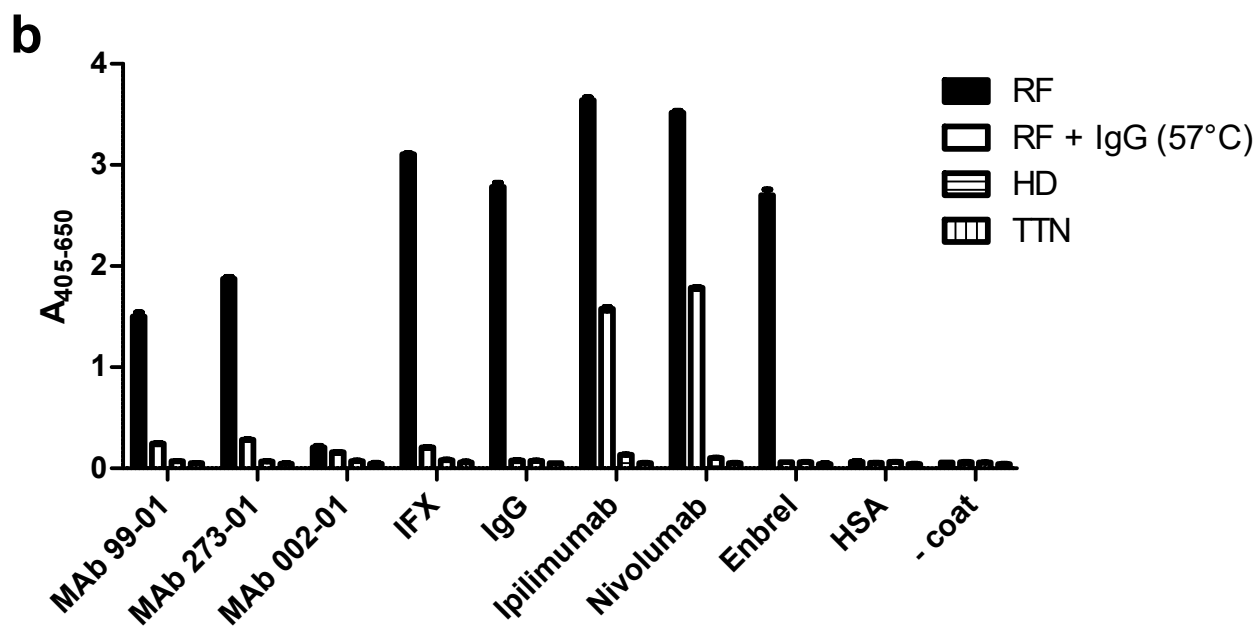

Supplement: S9 Fig — (a). Reactivity of RFs (IgM) with native (37 °) or heat-treated IFX (39 ° - 57 °C) in a bridging ELISA with IFX (non-heated) coated on the polystyrene surface of ELISA plate wells. (b). Reaction of RF IgM with IgG, different human therapeutic IgGs (IFX, Ipilimumab, Nivolumab), the TNFR-Fc biological drug Enbrel and three murine monoclonal antibodies (Mab) (02–01, 99–01, 273–01). Figures show means of double determinations and are from one representative experiment out of two. (PDF) [file pone.0217624.s009.pdf]

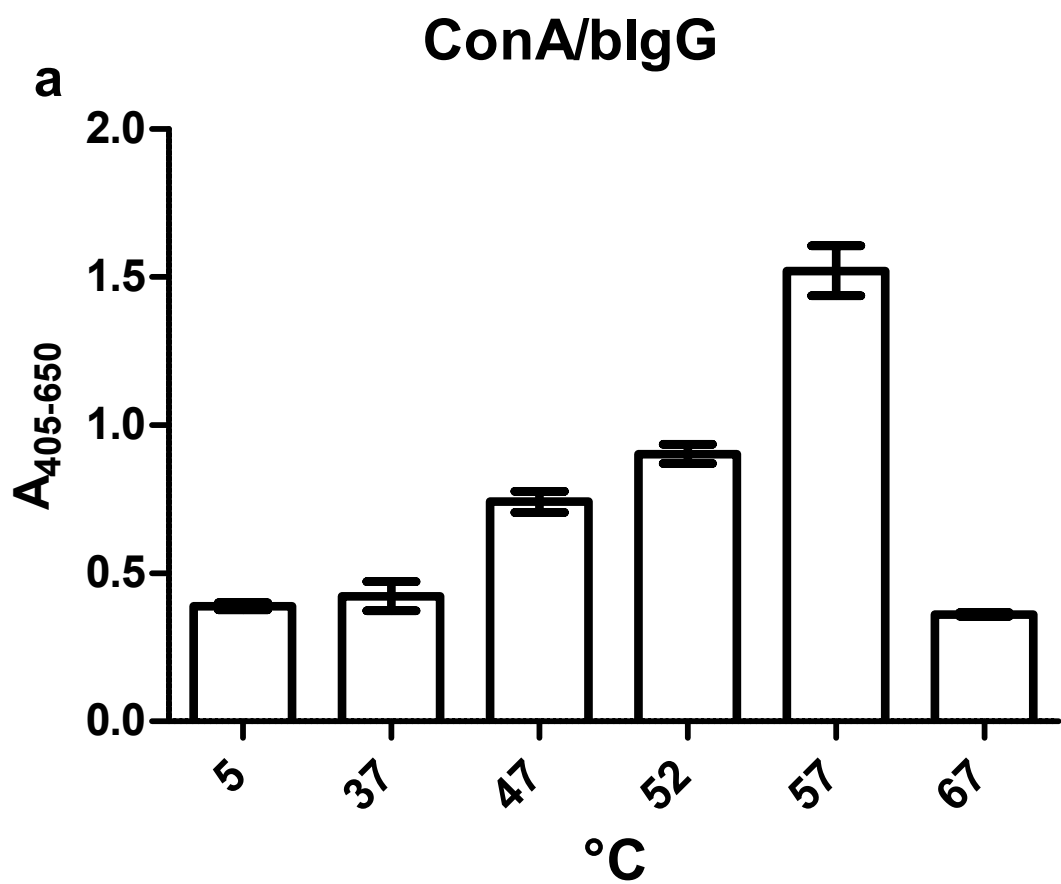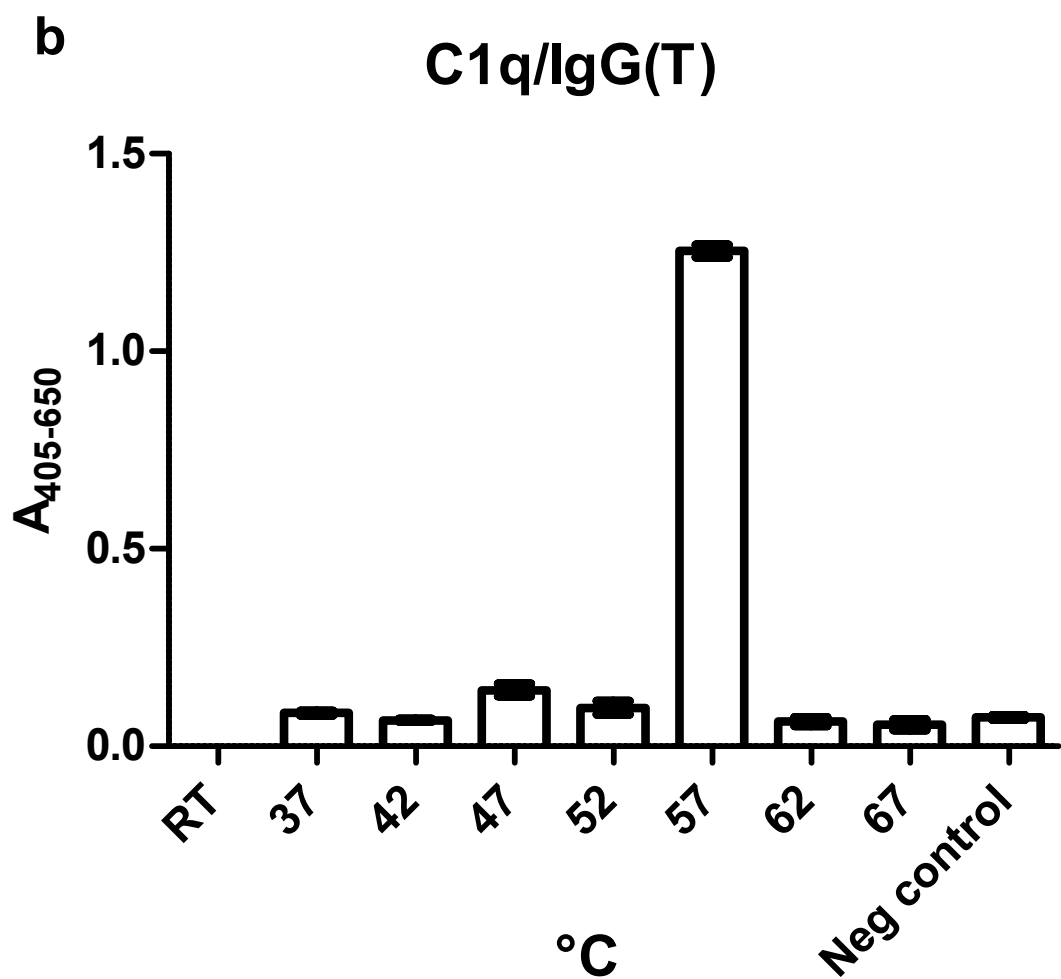

Supplement: S10 Fig — (a). Binding of native or heat-treated IgG to Concanavalin A. IgG pre-incubated at the indicated temperatures was incubated in ELISA plate wells coated with protein G and then tested for reaction with ConA (biotinylated, allowing detection with AP-conjugated streptavidin). (b). IgG conformational change upon heating allowing interaction with C1q. C1q was immobilized on the surface of ELISA wells and incubated with bIgG, which had been pre-incubated at the indicated temperatures. The interaction was maximal, when bIgG had been subjected to heating at 57 °C. Figures show means of double determinations and are from one representative experiment out of two. (PDF) [file pone.0217624.s010.pdf]

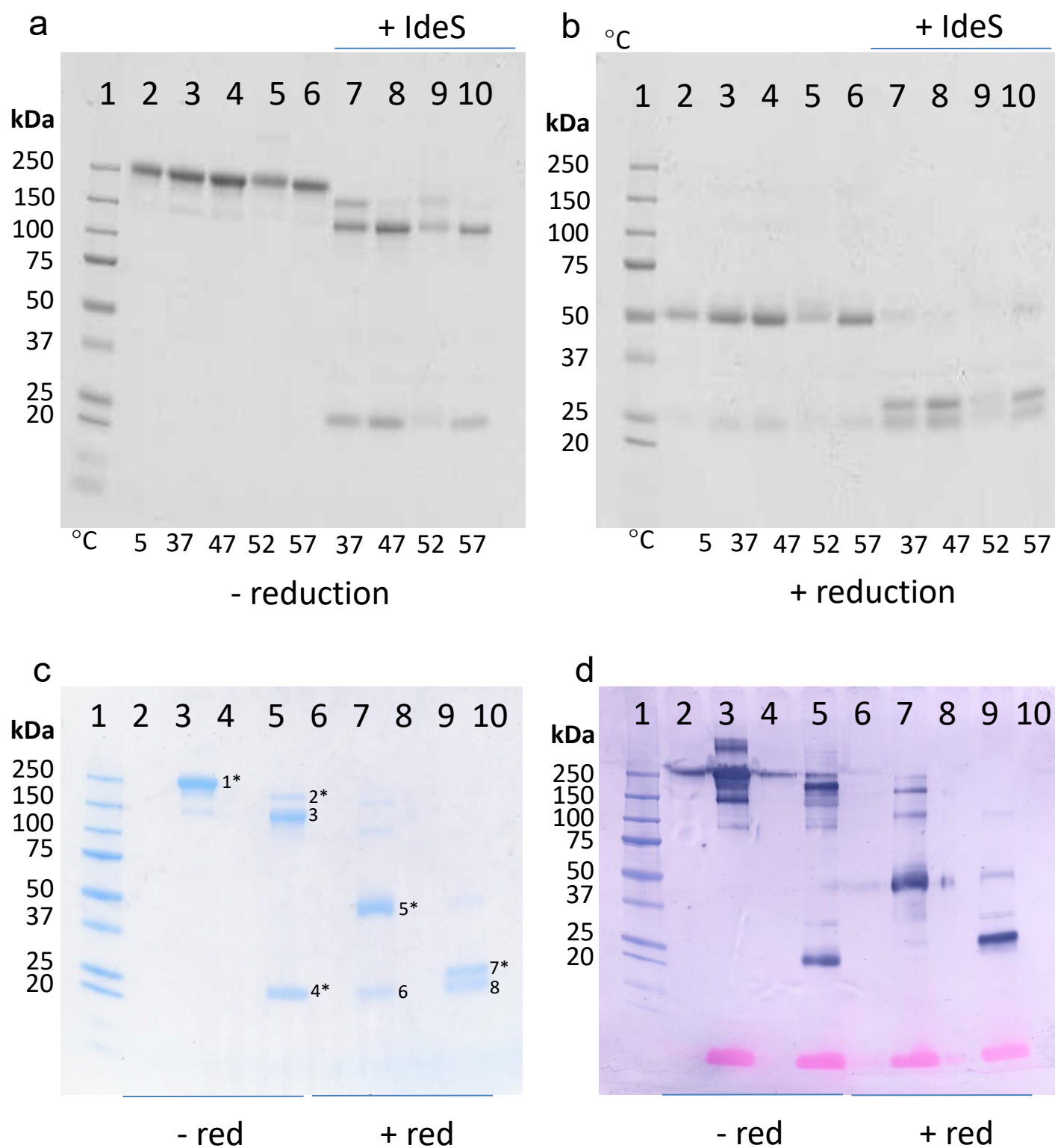

Supplement: S11 Fig — (a, b). SDS-PAGE analysis of IgG subjected to cleavage with the hinge-specific protease Ide S. IgG was preincubated at temperatures from 5 °C to 57 °C and then incubated with or without Ide S. Samples were analysed by SDS-PAGE on 4–20% gels without (a) and with (b) reduction with DTT. (c, d). SDS-PAGE (a) and Western immunoblotting (b) analysis of intact (lanes 3, 7) and IdeS-cleaved (lanes 5, 9) IgG with a pool of RF-IgM-positive sera. Note that strong reaction is seen with Fc-containing bands (*), while only a faint non-specific reaction is seen with F(ab’)2. Also note that the mobility of Fc is influenced by reduction. Figures are from one representative experiment out of two. Bands are assigned as follows; IgG (Fab2Fc)One-hinge-cleaved IgG (FabFc)Fab2FcHeavy chainLight chainFc (lower mobility than in lane 4 due to intact, non-reduced S-S bonds)Light chain, Variable heavy-CH1. (PDF) [file pone.0217624.s011.pdf]

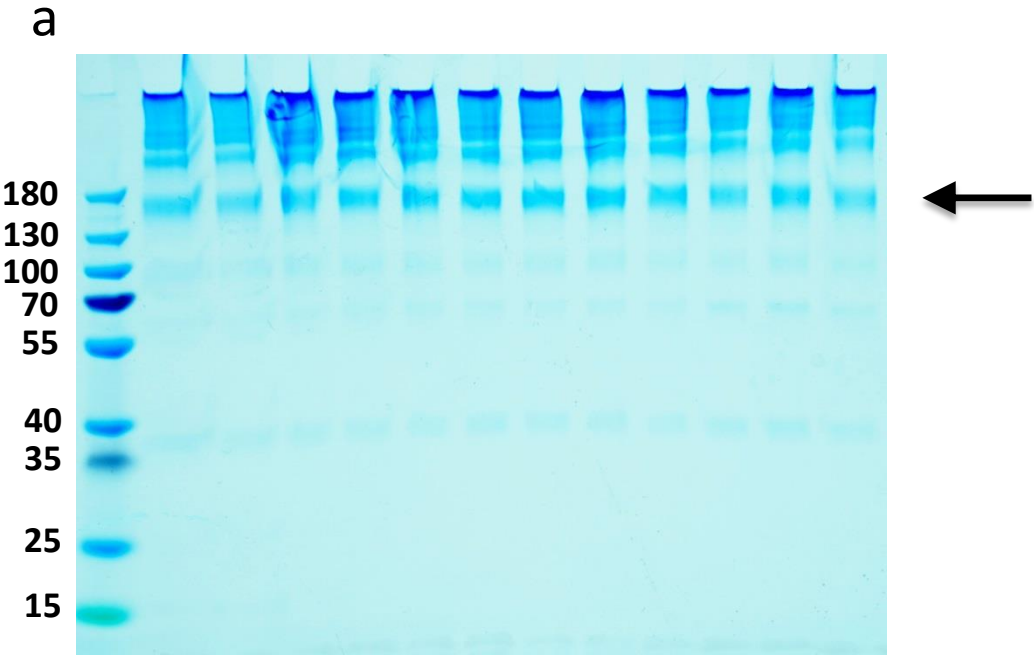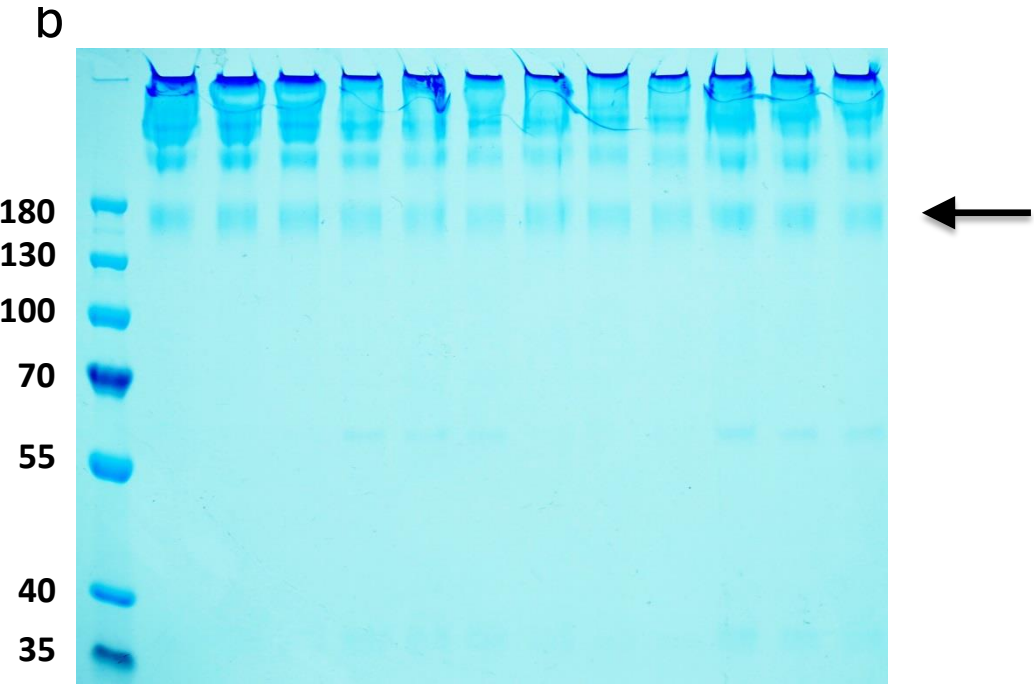

Supplement: S12 Fig — SDS-PAGE gels of BS3-crosslinked IFX in experiment 1 (0.5 μg/μL IFX) and experiment 2 (1μg/μL IFX), respectively. Both gels show a single band between 130–180 kDa markers, corresponding to monomeric crosslinked IFX molecules. These bands were excised for analysis. (PDF) [file pone.0217624.s012.pdf]

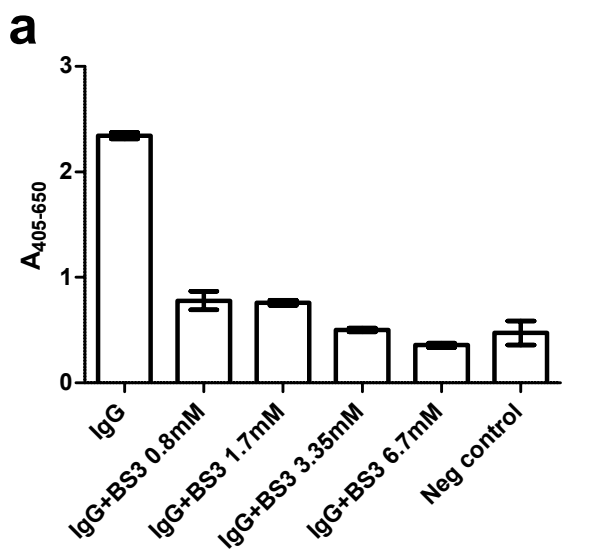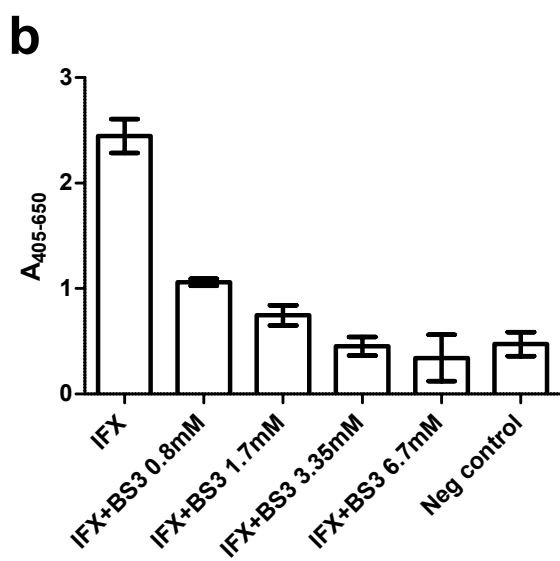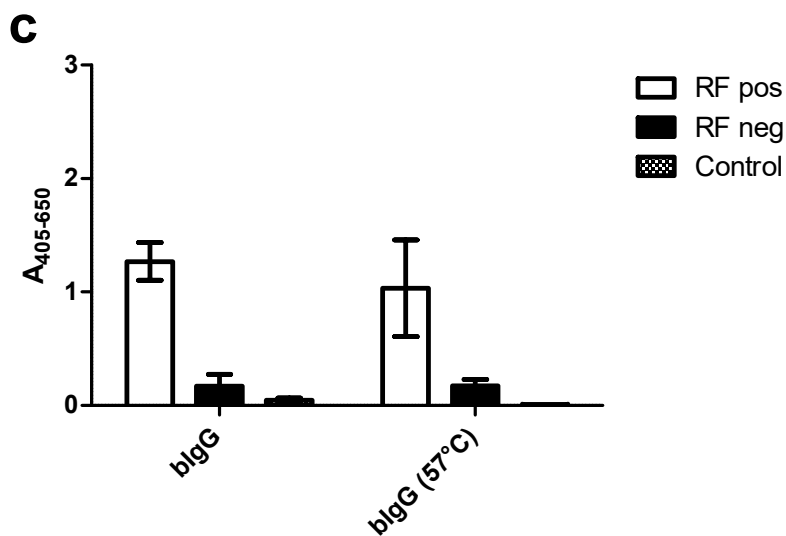

Supplement: S14 Fig — (a). Effect of bis-succinimidyl-suberate (BS3) cross-linking on RF IgM reaction with coated IFX as determined by ELISA. (b). Effect of BS3 cross-linking on RF IgM reaction with coated IgG as determined by ELISA. (c). Effect of biotinylation on RF IgM reaction with coated IgG/bIgG as determined by ELISA. The experiments were done by using the modified IFX/IgG as antigen in the routine RF IgM ELISA and figures show one representative experiment out of two. (PDF) [file pone.0217624.s014.pdf]
