# Supplementary material for: Immunoglobulin G structure and rheumatoid factor epitopes
Source: PLoS One. 2019 Jun 14;14(6):e0217624. doi: 10.1371/journal.pone.0217624 (PMC6568389; doi:10.1371/journal.pone.0217624)

prec. 3499.716

(INFLIXIMAB_HC) SKSINSATHYAESVK X (INFLIXIMAB_HC) DDSKSAVYLQMTDLR

VH-54 X VH-78

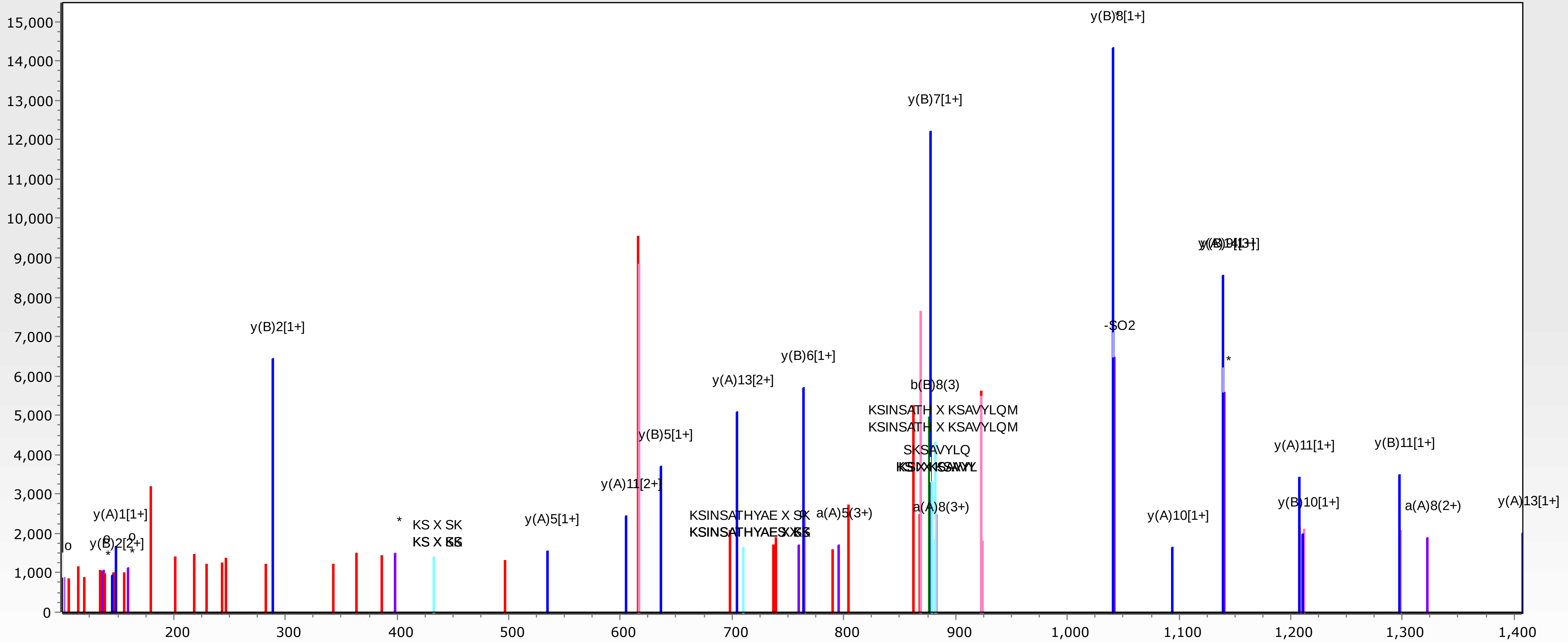

prec. 3225.73
(INFLIXIMAB_HC) GPSVFPLAPSSKSTSGGTAALGCLVK X (INFLIXIMAB_HC) KVEPK

CH1-136 X CH1-217

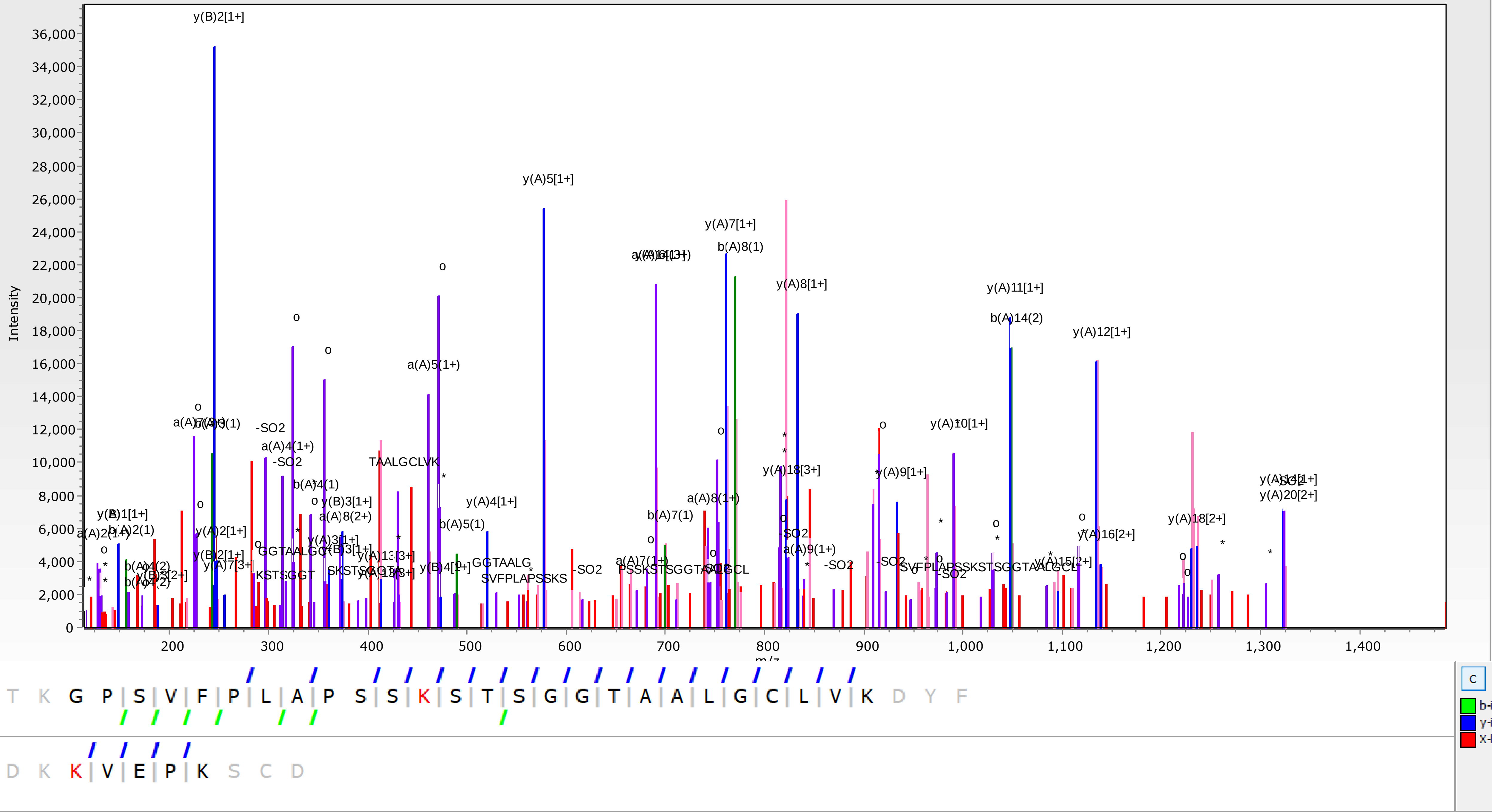

CH2-320 X CH2-343

prec. 3020.664
(INFLIXIMAB_HC) VVSVLTVLHQDWLNGKEYK X (INFLIXIMAB_HC) AKGQPR

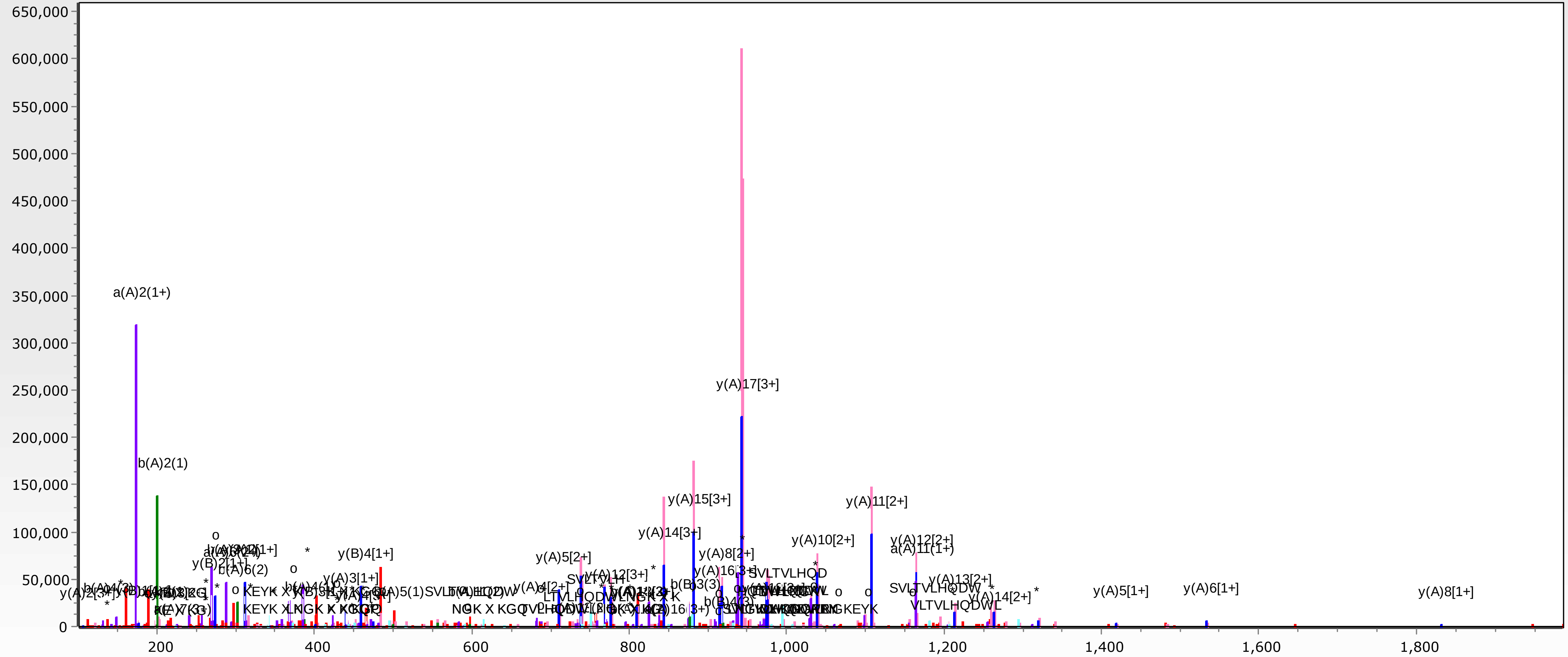

prec. 2060.199
(INFLIXIMAB_HC) ALPAPIEKTISK X (INFLIXIMAB_HC) AKGQPR

CH2-337 X CH2-343

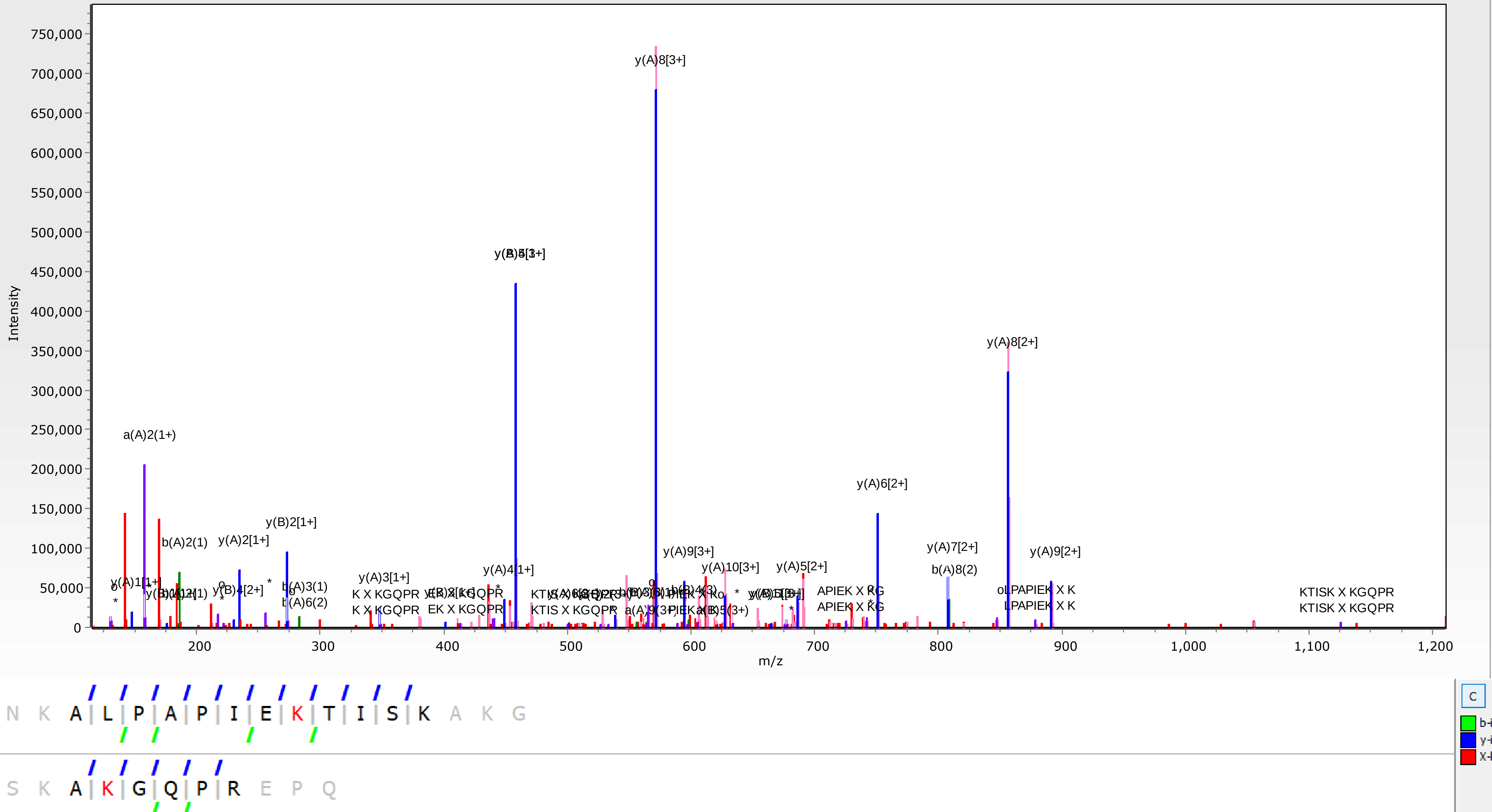

prec. 3673.953
(INFLIXIMAB__LC) DILLTQSPAILSVSPGER X (INFLIXIMAB_HC) QSPEKGLEWVAEIR

VL-1 X VH-43

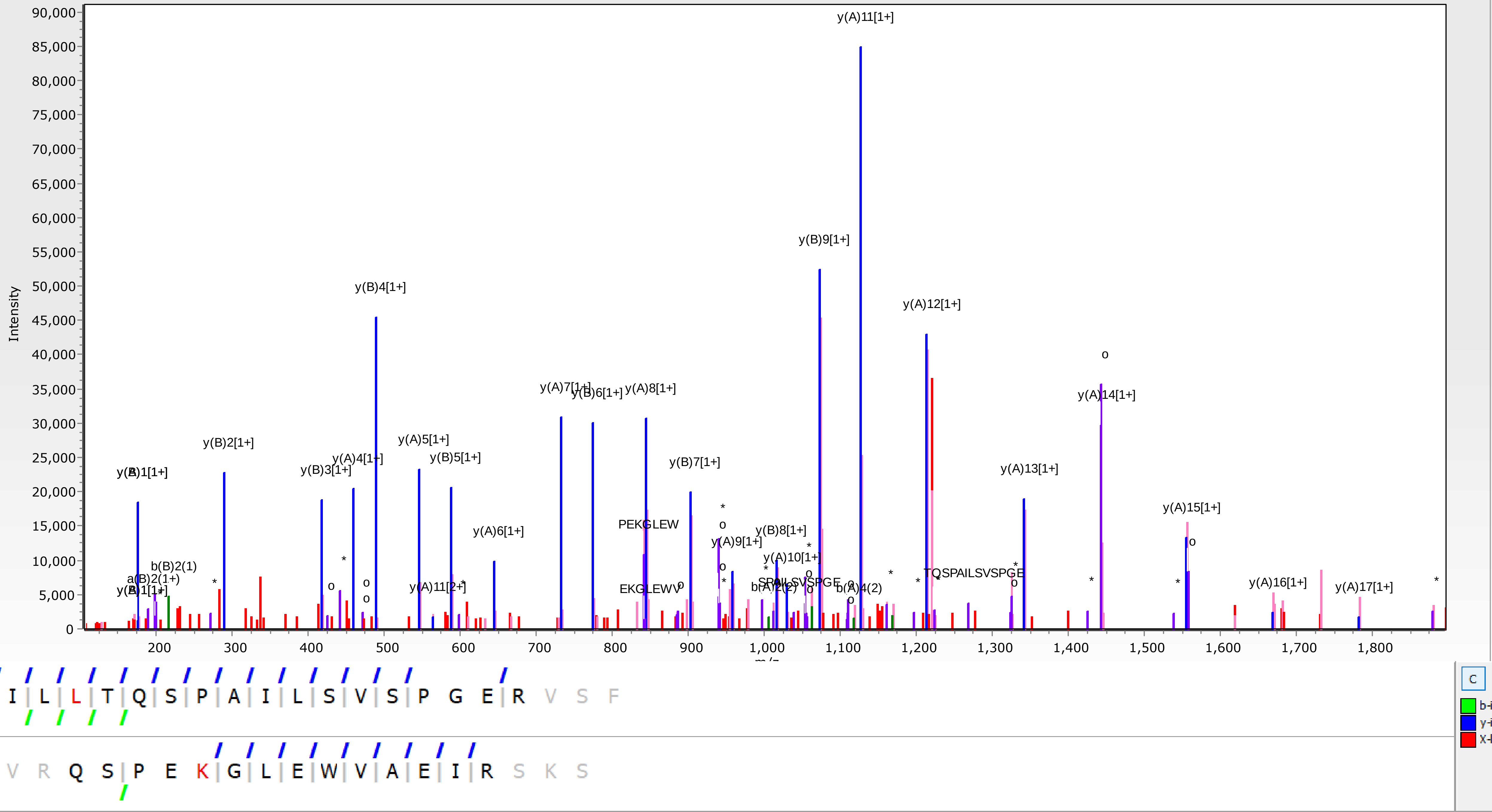

prec. 3653.921
(INFLIXIMAB__LC) DILLTQSPAILSVSPGER X (INFLIXIMAB_HC) SKSINSATHYAESVK

VL-1 X VH-54

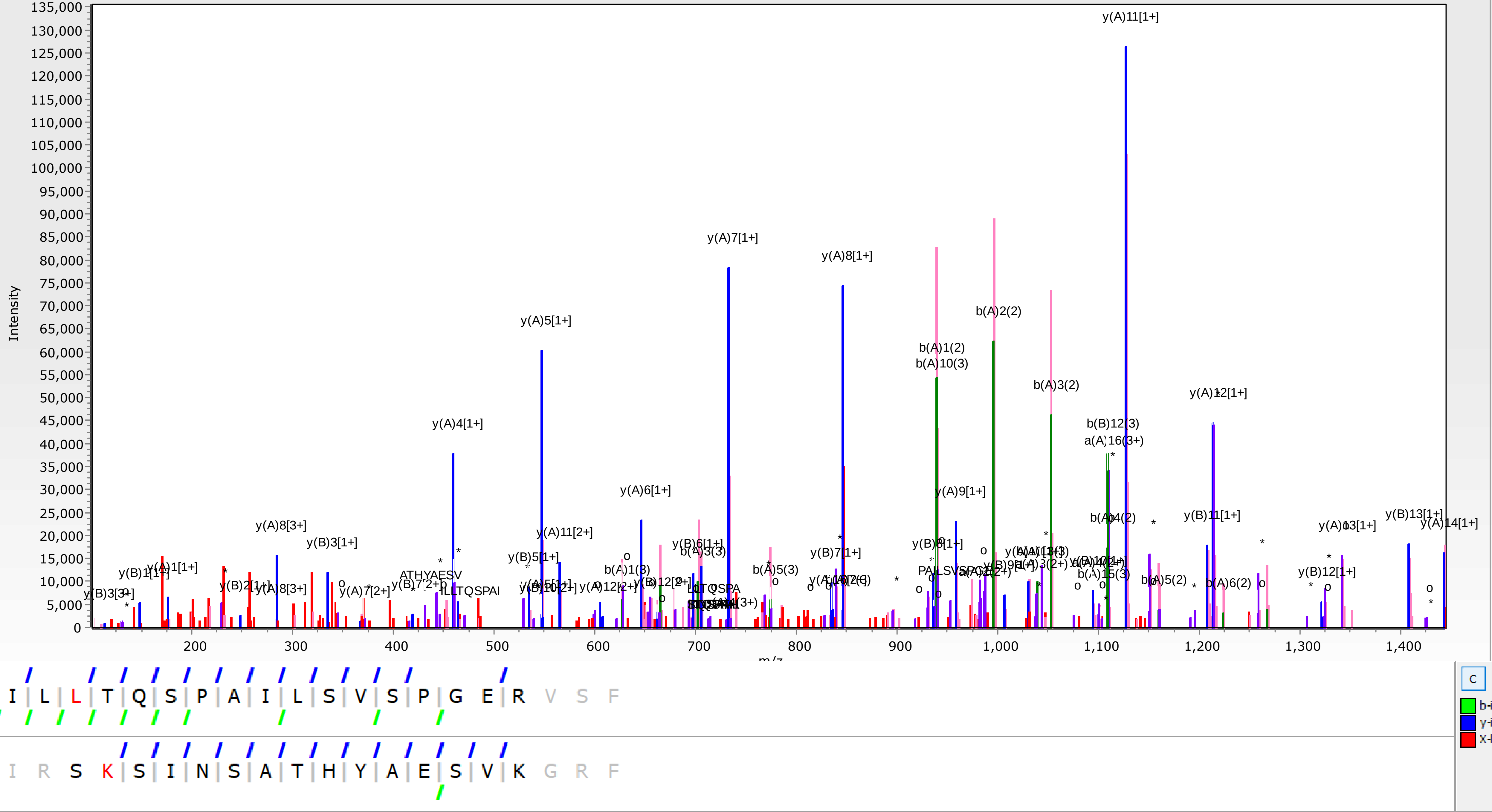

prec. 4023.12

(INFLIXIMAB__LC) DILLTQSPAILSVSPGER X (INFLIXIMAB_HC) SKSINSATHYAESVKGR

VL-1 X VH-54

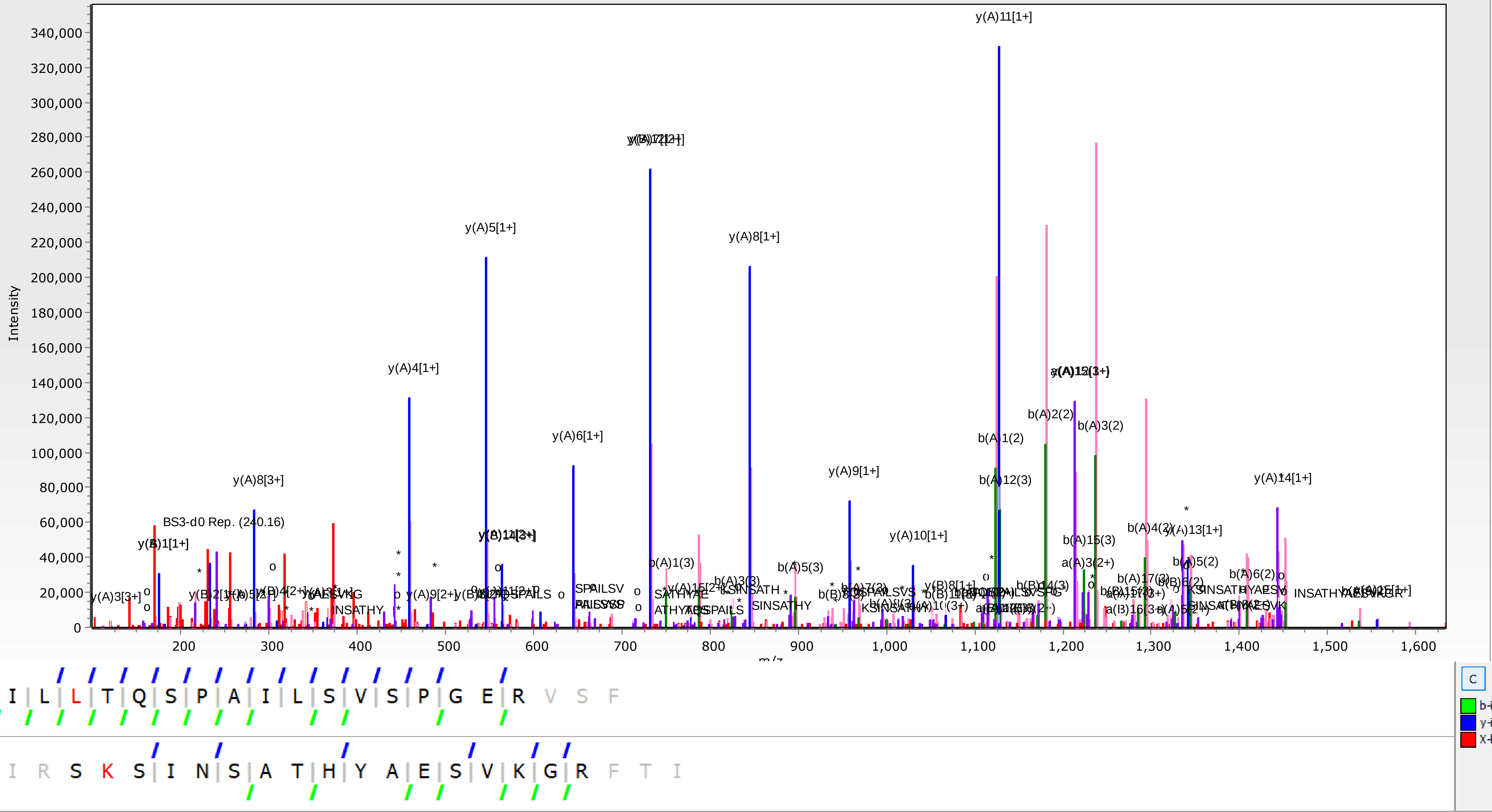

prec. 4022.155

(INFLIXIMAB__LC) DILLTQSPAILSVSPGER X (INFLIXIMAB_HC) SKSINSATHYAESVKGR

VL-1 X VH-54

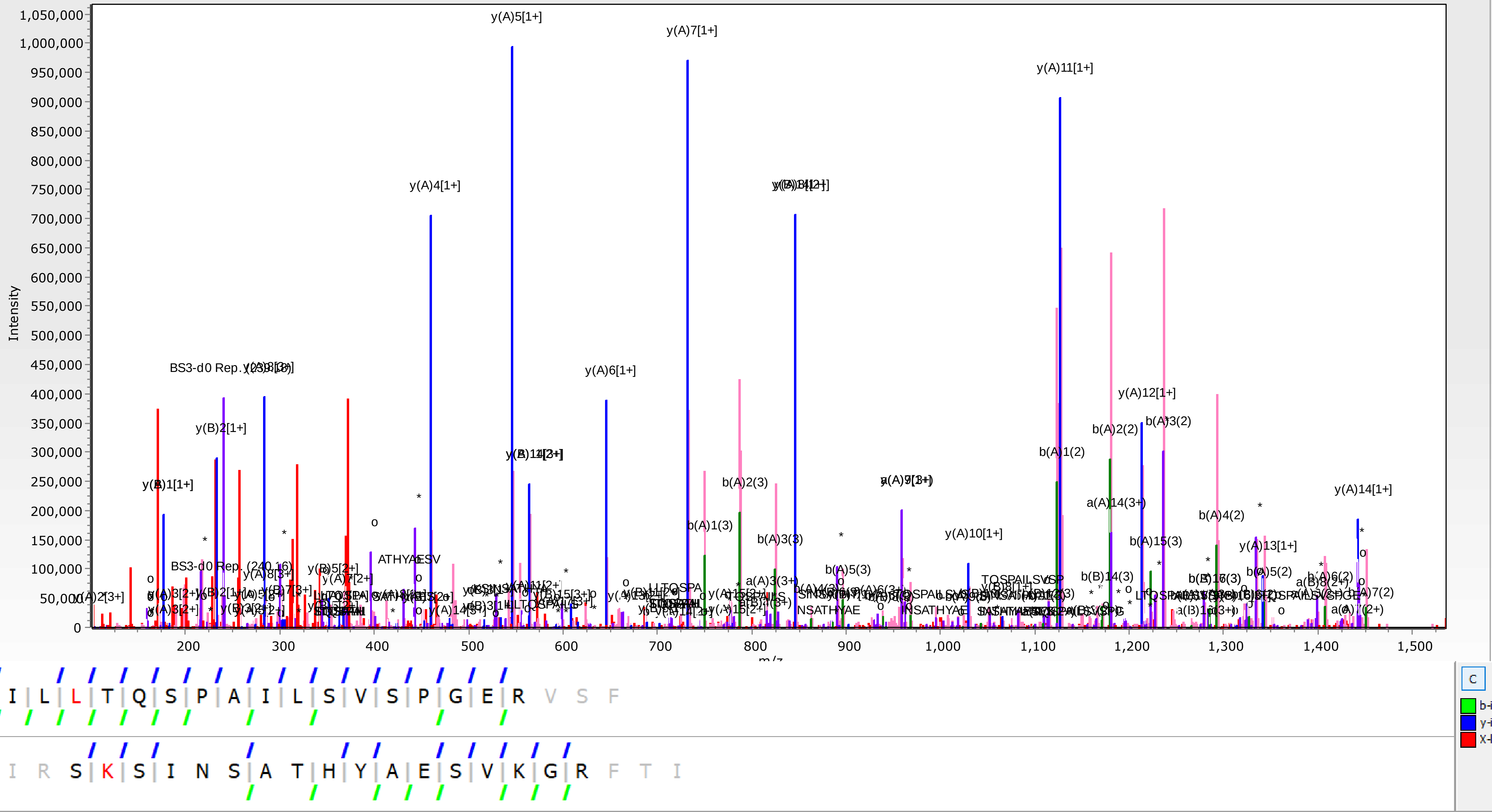

prec. 3651.919
(INFLIXIMAB__LC) DILLTQSPAILSVSPGER X (INFLIXIMAB_HC) SINSATHYAESVKGR

VL-1 X VH-67

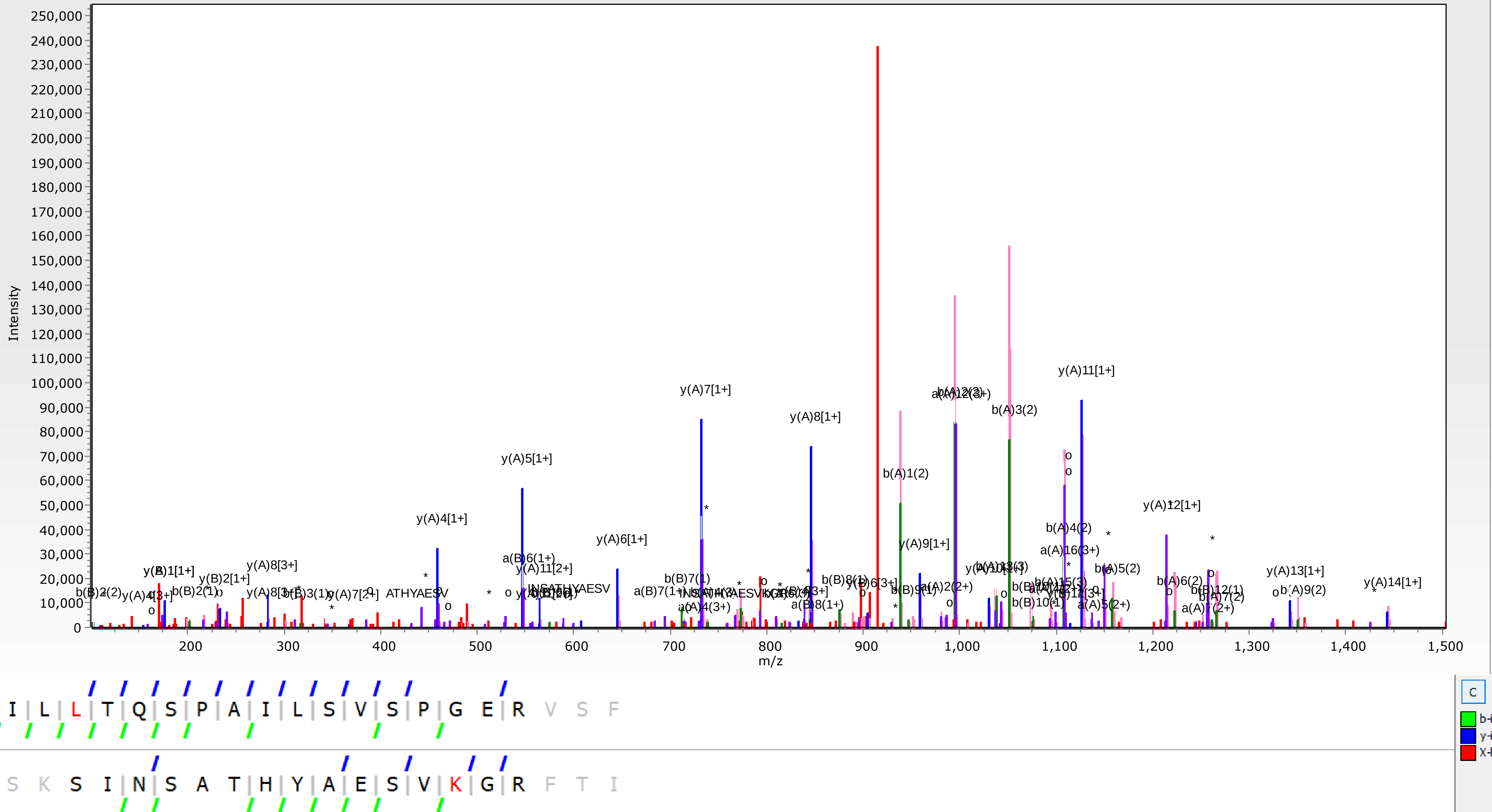

Overlength crosslinks

VH-54 X CH3-417

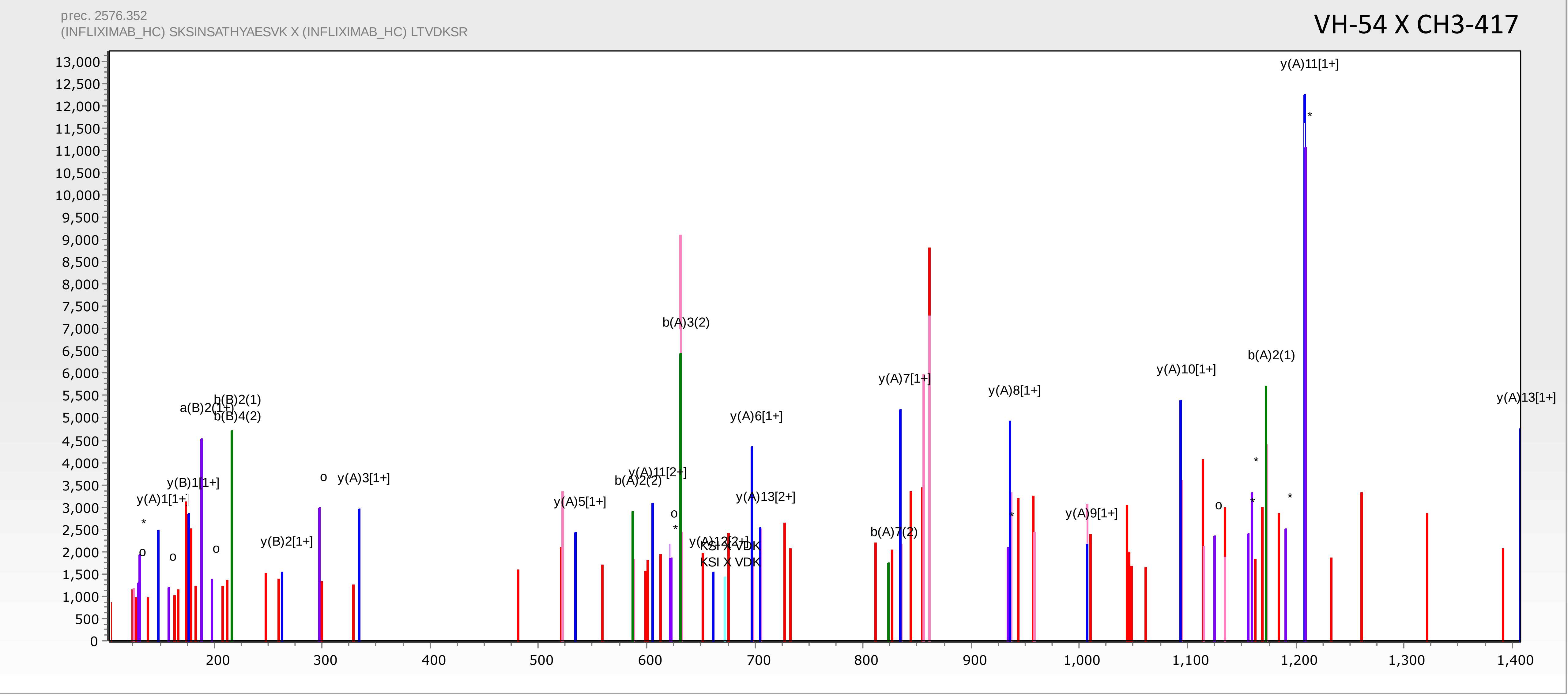

prec. 3892.102
(INFLIXIMAB_HC) GPSVFPLAPSSKSTSGGTAALGCLVK X (INFLIXIMAB_HC) VSNKALPAPIEK

CH1-136 X CH2-329

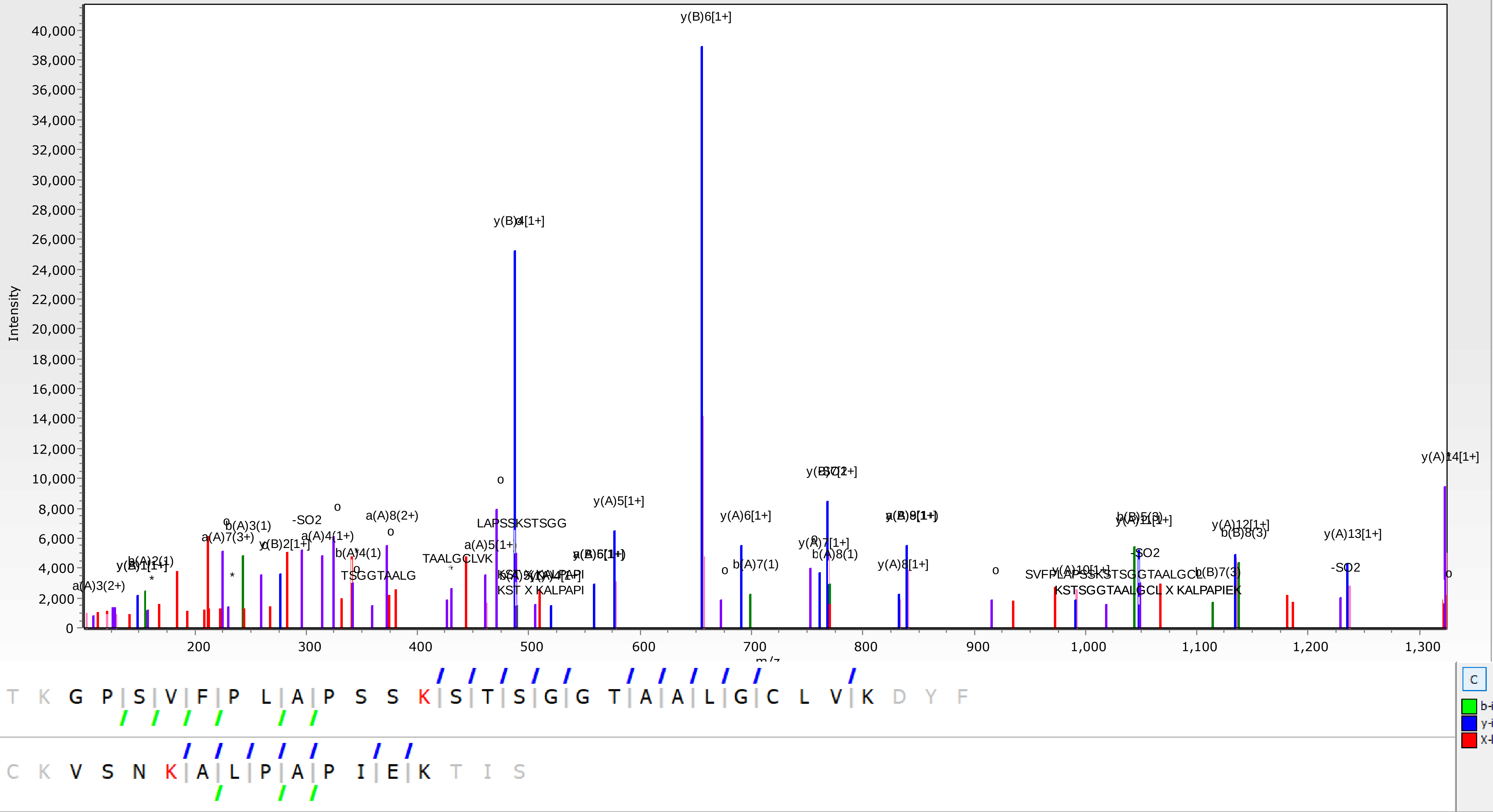

prec. 3281.732
(INFLIXIMAB_HC) GPSVFPLAPSSKSTSGGTAALGCLVK X (INFLIXIMAB_HC) AKGQPR

CH1-136 X CH3-343

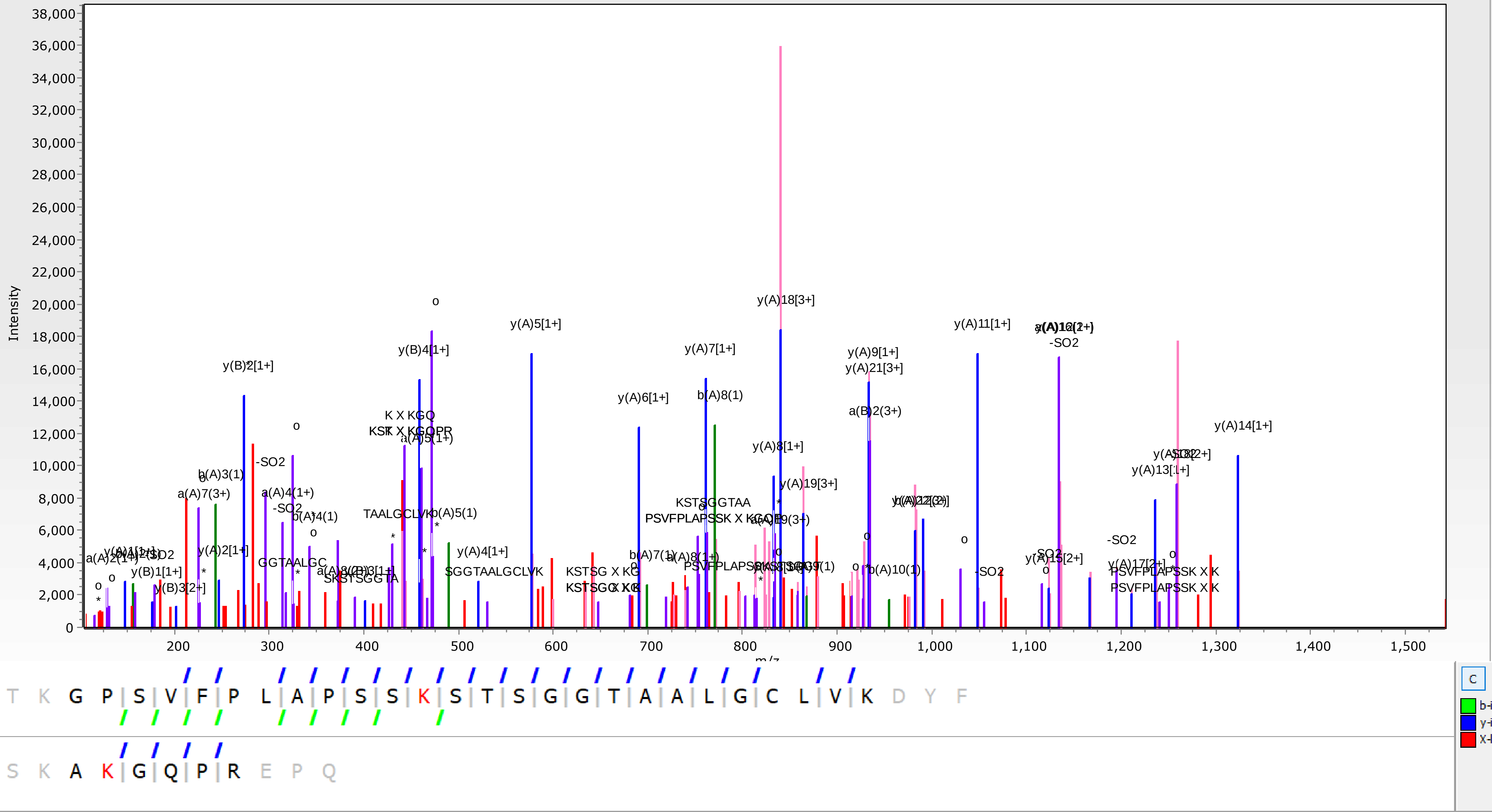

prec. 2712.361
(INFLIXIMAB_HC) LTVDKSR X (INFLIXIMAB_HC) DDSKSAVYLQMTDLR

CH3-417 X VH-78

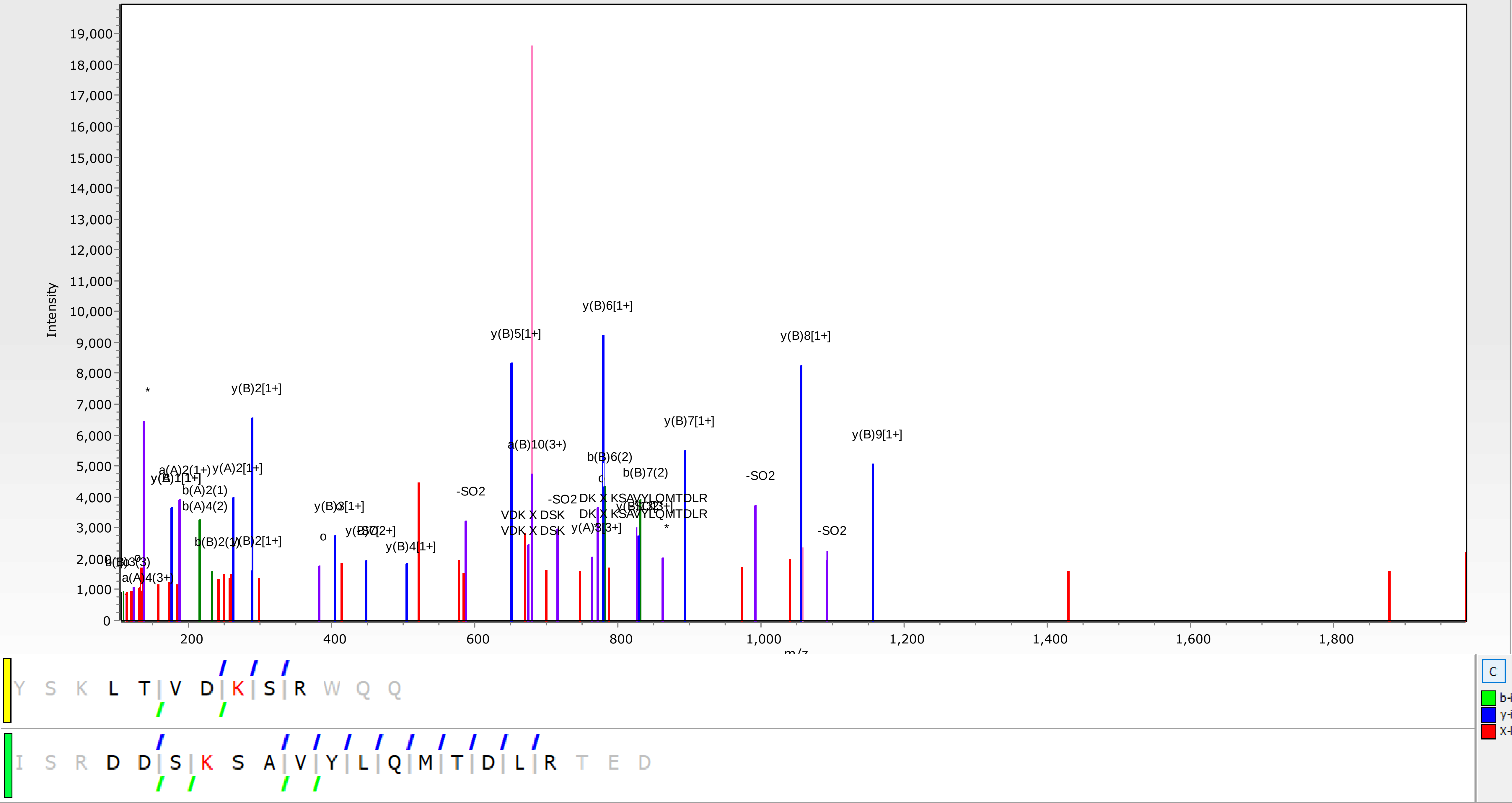

Supplement: S13 Fig — In the spectra, y- and b-fragment ions are presented in blue and green, respectively. Purple peaks represent neutral losses, pink peaks represents isotope ions, and red peaks are unassigned ions. Below each spectrum is the annotated sequences of peptide A (top) and peptide B (bottom). Blue and green marks above and below the sequence indicates observed y- and b-ions, respectively. The cross-linked residues are marked in red and their sequence numbers and domains are shown in the top right corner. (PDF) [file pone.0217624.s013.pdf]
